# Synthesis of Azo-Substituted Benzoxazin-4-ones by Base-Mediated Addition of Diazenyl Anions to Isatoic Anhydrides

**DOI:** 10.1021/acs.orglett.6c02115

**Published:** 2026-06-27

**Authors:** Wolfgang Obermayer, Elisabeth Irran, Martin Oestreich

**Affiliations:** Institut für Chemie, 26524 Technische Universität Berlin, Strasse des 17. Juni 115, 10623 Berlin, Germany

## Abstract

A two-step synthesis of 4*H*-benzo­[*d*]­[1,3]­oxazin-4-ones bearing an azo unit at C2 from isatoic anhydrides and silylated diazenes is reported. The key step is the diazenylation of an isocyanate formed in situ by base-mediated ring opening of the isatoic anhydride. The addition of the elusive diazenyl anion proceeds *without* the loss of dinitrogen in the basic reaction medium. The benzoxazinone motif is subsequently established by cyclization with the Mukaiyama reagent.

## Graphical abstract

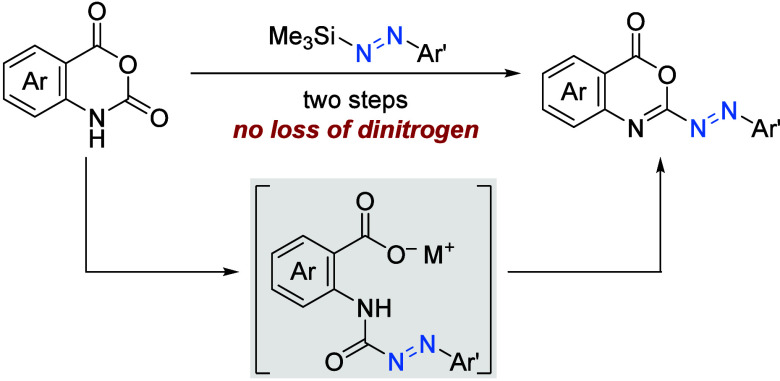

Benzoxazines are an important class of bioactive compounds found in a large number of pharmaceutically relevant molecules and drugs (Figure 1).^1^ For example, Cetilistat^2^ is a drug for the treatment of obesity and Etifoxine is an anxiolytic drug.^3^ While the synthesis of various 2-aryl/alkyl- and 2-amino-substituted benzoxazin-4-ones is largely established,^1^ there are no methods available for the synthesis of azo-substituted benzoxazinones. However, azo compounds display interesting pharmacological^4–6^ and photochemical^7–9^ properties and are used in pharmaceuticals and dyes and have been applied as molecular photoswitches. Hence, the combination of an azo unit with the benzoxazinone motif could lead to the discovery of drugs with novel pharmacological or photochemcial properties.

**Figure 1:**
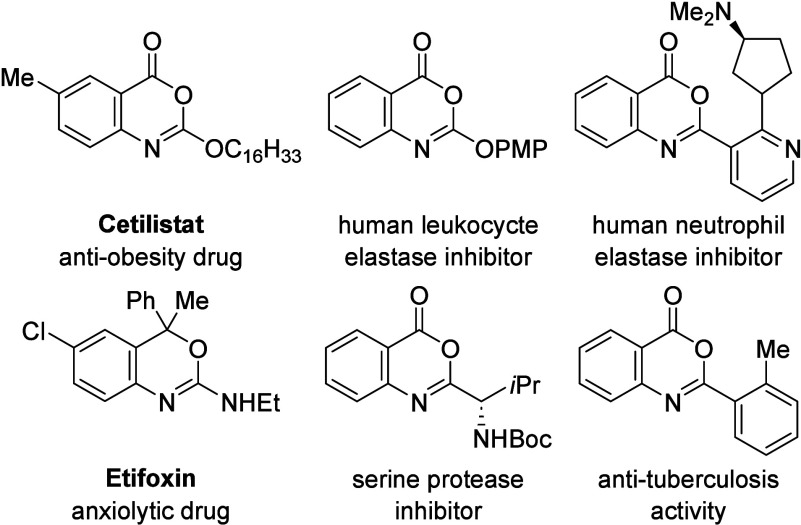
Examples of biologically active benzoxazines.

In contrast to the synthesis of symmetric azobenzenes, the synthesis of nonsymmetric azo compounds,^10^ especially those with one or two heterocyclic residues,^11,12^ remains a challenge. Our laboratory recently developed a palladium-catalyzed diazenylation of (hetero)­aryl (pseudo)­halides and *N*-silylated, aryl-substituted diazenes as masked diazenyl anions (Scheme 1A).^13–15^ Remarkably, *no loss of di* *nitrogen* was observed in this reaction, which is rationalized by a two-step transmetalation not involving the intermediacy of a free diazenyl anion.^16^ In previously published transition-metal-free reactions, diazenyl anions underwent rapid denitrogenation and formed the corresponding carbon nucleophiles,^17–21^ except for one example where alkyl-substituted diazenyl anion precursors were reacted with aldehydes (Scheme 1B).^22–24^ Yet, aryl-substituted diazenyl anions have never been coupled to electrophiles and have always released dinitrogen. When we extended this formal aryldiazenyl-anion chemistry to isatoic anhydrides as potential coupling partners, however, we found that no denitrogenation is observed even in the abscence of a transition-metal catalyst. This is a remarkable observation as diazenyl anions are fleeting intermediates prone to rapid fragmentation under dinitrogen extrusion.^25^ This prompted us to develop a transition-metal-free protocol for the synthesis of azocarboxamides under mild conditions, which are cyclized in a second step to form 2-azo-substituted benzoxazin-4-ones (Scheme 1C).

**Scheme 1:**
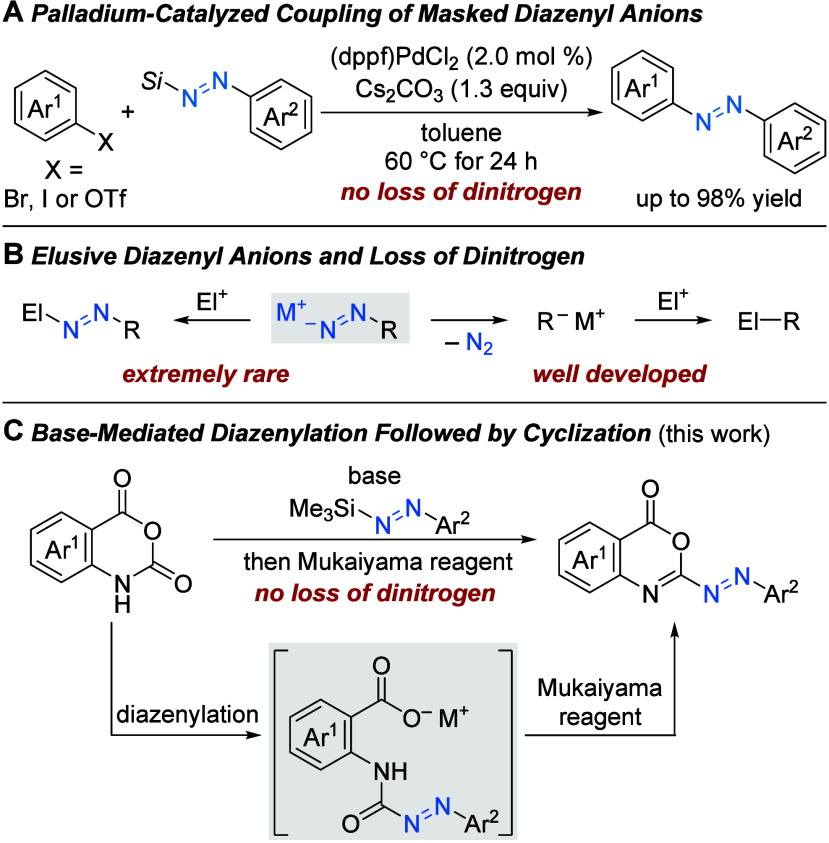
Azobenzene Formation by Palladium-Catalyzed or Base-Mediated Diazenylation

We started our optimization of the ring-opening step by reacting isatoic anhydride (**1a**) with *N*-phenyl-*N*′-trimethylsilyldiazene (**2a**) in 1,2-dichlorobenzene using sodium carbonate as the base (Table 1, entry 1). The azocarboxamide was formed in 87% yield and was afforded in the form of its carboxylate **3aa**·Na after methanolysis. Changing the base to the more nucleophilic sodium *tert*-butoxide resulted in no product formation (entry 2) while replacing the sodium cation in the carbonate base with lithium, potassium, or cesium resulted in diminished yields (entries 3–5). We continued testing the effect of various solvents on the reaction outcome (entries 6–8). Using chlorobenzene resulted in a high yield (entry 6), but nonpolar toluene and polar, Lewis basic THF reduced the yield significantly (entries 7 and 8). Finally, reducing the excess of diazene from 2.0 to 1.7 equiv did not result in a notable change in yield whereas further decreasing to 1.4 equiv was detrimental to product formation (entries 9 and 10). It is important to note that no formation of the denitrogenation product **4aa**·M was observed which would have resulted from an attack of the corresponding aryl anion to the starting material (see Scheme 1B, right pathway). Additionally, we conducted a control experiment with no base and, as expected, no product was formed (entry 11); instead, both the silyldiazene **2a** and the anhydride **1a** decomposed. We continued by optimizing the cyclization reaction (Table S2). For the identification of coupling reagents and optimization of the solvent system, we used the free carboxylic acid **3aa**·H, afforded by washing of **3aa**·Na with 1 N HCl, and found that the use of the Mukaiyama reagent in a mixture of CH_2_Cl_2_ and MeCN results in near-quantitative yields (Scheme 2).

**Scheme 2:**
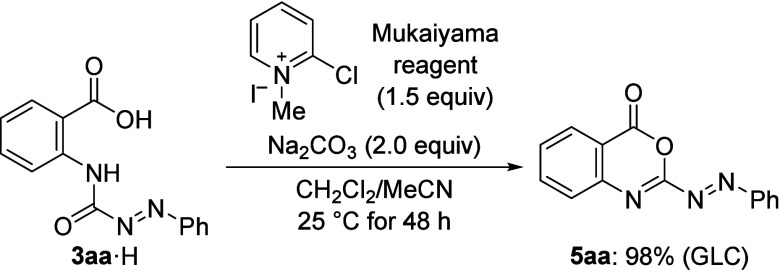
Ring Closure Mediated by the Mukaiyama Reagent

**Table 1:**
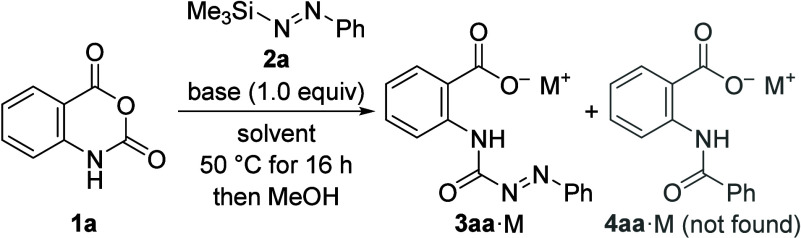
Optimization of the Ring-Opening Diazenylation of Isatoic Anhydride

entry	base (equiv)	solvent	**2a** (equiv)	yield of **3aa**·M (%)^a^
1	Na_2_CO_3_ (1.0)	1,2-Cl_2_C_6_H_4_	2.0	87
2	NaO*t*Bu (1.0)	1,2-Cl_2_C_6_H_4_	2.0	0
3	Li_2_CO_3_ (1.0)	1,2-Cl_2_C_6_H_4_	2.0	70
4	K_2_CO_3_ (1.0)	1,2-Cl_2_C_6_H_4_	2.0	71
5	Cs_2_CO_3_ (1.0)	1,2-Cl_2_C_6_H_4_	2.0	56
6	Na_2_CO_3_ (1.0)	ClC_6_H_5_	2.0	94
7	Na_2_CO_3_ (1.0)	toluene	2.0	79
8	Na_2_CO_3_ (1.0)	THF	2.0	70
9	Na_2_CO_3_ (1.0)	ClC_6_H_5_	1.7	91
10	Na_2_CO_3_ (1.0)	ClC_6_H_5_	1.4	63
11		ClC_6_H_5_	1.7	0

a Yields were determined by quantitative ^1^H NMR spectroscopy using 1,3,5-trimethoxybenzene as an internal standard.

With the optimized two-step procedure in hands, we investigated the reaction scope (Scheme 3). We began by isolating our model product **5aa** and obtained 70% yield over two steps. We continued by testing the influence of electron-donating and electron-withdrawing groups in different positions on the benzoxazin-4-one ring. Substitution in the 5, 6, and 7 positions with electron-donating groups (**5ba**, **5da**, **5ga**) resulted in good yields while isatoic anhydrides substituted with electron-withdrawing groups required *n*-hexane as the solvent and a reduced temperature in the ring-opening step to form the products in acceptable yields. Using these altered conditions, chloro (**5ca**, **5ea**, **5ha**) and fluoro (**5ja**) substituents were accepted well by our system but the electron-deficient 7-nitro-substituted benzoxazine-4-one **5ja** proved to be unstable on silica gel and alumina and decomposed during column chromatography. Furthermore, substitution at C8 prevented formation of the ring-opened product, and the carboxamidonaphthoate **3ma**·Na proved to be insoluble in organic solvents (gray box). Finally, the diazenyl residue was varied. Substitution of the diazenyl residue with electron-donating and moderately strongly electron-withdrawing groups resulted in good yields and substitution with strongly electron-withdrawing groups led to a decrease in yield.

**Scheme 3:**
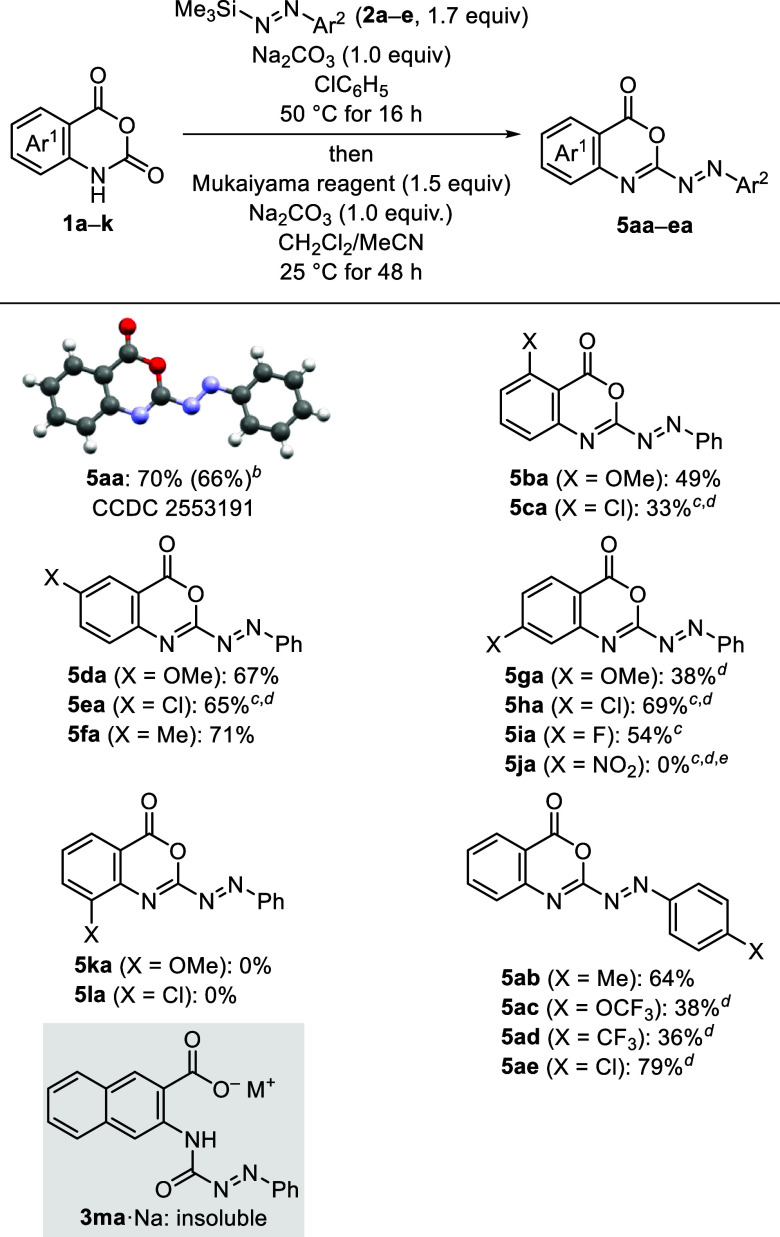
Investigation of the Substrate Scope^a^

a Unless otherwise stated, all reactions were performed on a 0.30 mmol scale. Yields are of isolated products after two steps. Isolation was performed by automated flash column chromatography on silica gel.
b Yield in parentheses was obtained on a 1.0 mmol scale.
c The ring-opening reaction was performed in *n*-hexane at room temperature.
d For diazenecarboxamidobenzoates **3**·Na which exhibited low solubility in CH_2_Cl_2_/MeCN, an acidic workup was performed, and the free acid **3**·H was cyclized using 2.0 equiv of triethylamine as the base in the cyclization step.
e Decomposed during column chromatography.

We think that the ring-opening reaction proceeds through the mechanism outlined in Scheme 4A. In the first step, isatoic anhydride (**1a**) is deprotonated at the NH-group followed by ring opening to 2-isocyanatobenzoate **6a**·Na.^26^ The thus-generated carboxylate anion **6a**·Na attacks the silicon atom of the silyldiazene **2a** to initially arrive at a pentacoordinate Lewis adduct (not shown). The increased Lewis acidity of that silicon atom as well as the possibility of forming a six-membered chelate likely allows the formation of the hexacoordinate species **7aa**·Na where the lone pair of the isocyanate nitrogen atom is coordinated to the silicon atom (gray box). This brings the diazenyl nucleophile in close proximity to the electrophilic isocyanate carbon atom, hence enabling its transfer without loss of dinitrogen. By this, the azo group is introduced, and the silyl ester in **8aa**·Na is subsequently cleaved with methanol. To confirm the deprotonation step, we subjected *N*-methyl isatoic anhydride (**9**) to the reaction conditions as this compound does not possess an acidic proton on the nitrogen atom (Scheme 4B). Upon analysis of the reaction mixture by LCMS, only methyl 2-(methylamino)­benzoate and azobenzene were detected. Azobenzene resulted from the decomposition of the silyldiazene in methanol^27^ (see the Supporting Information) while 2-(methylamino)­benzoate was formed by methanolysis of the anhydride. This proves that no reaction occurred between the two starting materials.

**Scheme 4:**
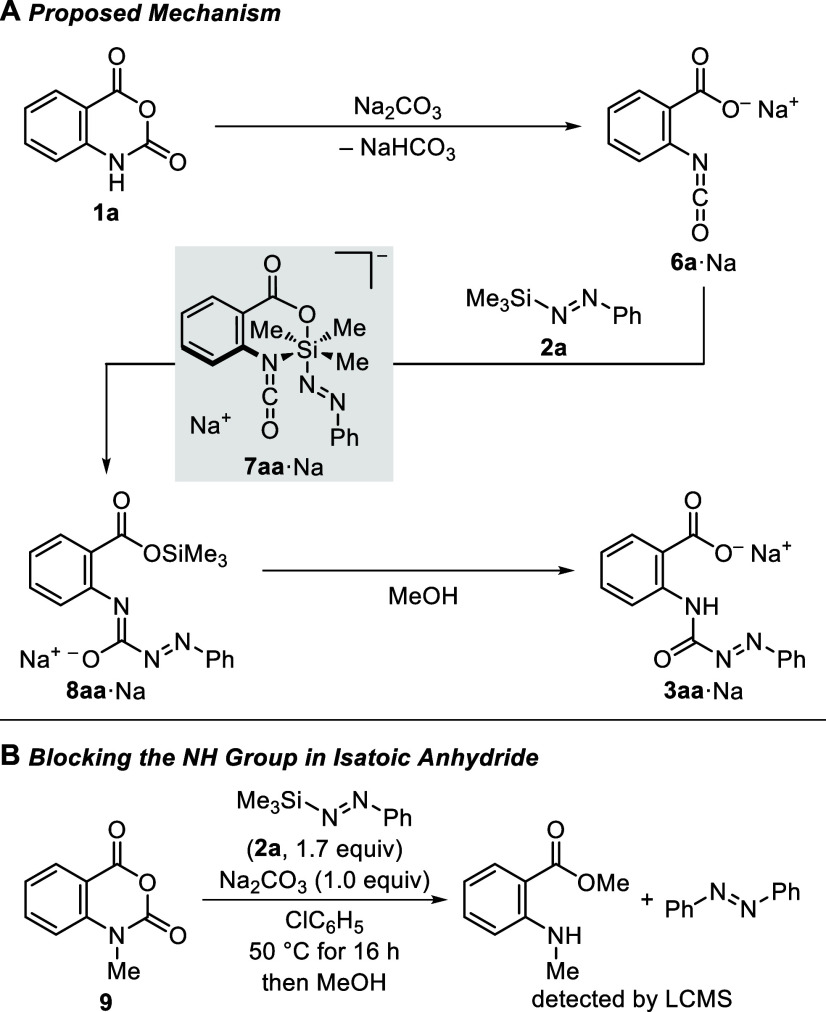
Mechanistic Analysis

In summary, we developed a method for the synthesis of azo-substituted benzoxazin-4-ones under mild conditions using a ring-opening/ring-closing reaction sequence. The azo-substituted products were afforded in satisfactory yields. This became possible since the diazenyl anion was selectively transferred to the starting material and did not undergo denitrogenation to form the corresponding aryl anion.

## Supplementary Material



## Data Availability

The data underlying this study are available in the published article and its Supporting Information.

## Notes

General procedures, experimental details, characterization and spectral data for all new compounds, and X-ray crystallographic data (PDF)
