# Supplementary material for: Synthesis of Azo-Substituted Benzoxazin-4-ones by Base-Mediated Addition of Diazenyl Anions to Isatoic Anhydrides
Source: Org Lett. 2026 Jun 27;28(27):8617–20. doi: 10.1021/acs.orglett.6c02115 (PMC13366685; doi:10.1021/acs.orglett.6c02115)
Supplement: Supplementary file 1 [file ol6c02115_si_001.pdf]

Synthesis of Azo-Substituted Benzoxazin-4-ones by Base-Mediated Addition of Diazenyl Anions to Isatoic Anhydrides

Wolfgang Obermayer, Elisabeth Irran, and Martin Oestreich*

Institut für Chemie, Technische Universität Berlin,

Straße des 17. Juni 115, 10623 Berlin, Germany

martin.oestreich@tu-berlin.de

Supporting Information

Table of Contents

1 General Information 3

2 General Procedures 5

2.1 General Procedure for the Synthesis of Azo-Substituted Benzoxazine-4-ones using Sodium Carbonate as the Base in the Cyclization Step (**GP 1**) 5

2.2 General Procedure for the Synthesis of Azo-Substituted Benzoxazine-4-ones using Triethylamine as the Base in the Cyclization Step (**GP 2**) 5

2.3 Optimization of the Ring Opening Reaction 6

2.4 Optimization of the Cyclization Reaction 7

3 Experimental Details for the Synthesis of (*E*)-2-(Phenyldiazenecarboxamido)-benzoic Acid (**3aa**·H) 9

4 Experimental Details for the Synthesis of Azo-Substituted Benzoxazinones...... 10

5 Synthesis of (*E*)-2-(Phenyldiazenyl)-4*H*-benzo[d][1,3]oxazin-4-one (**5aa**) on a 1.0 mmol Scale 19

6 Experimental Details for the Synthesis of *N*-Methylisatoic Anhydride (**9**) 20

7 Decomposition of *N*-Phenyl-*N′*-trimethylsilyldiazene **2a** in MeOH 21

8 Crystallographic Data 22

9 UV-Vis Spectrum of (*E*)-2-(Phenyldiazenyl)-4*H*-benzo[d][1,3]oxazin-4-one (**5aa**) 24

10 NMR Spectra 25

11 References 42

1 General Information

Unless otherwise stated, all reactions were performed in flame-dried glassware in an argon-filled *MB-Labstar* glovebox (O_2 < 0.5 ppm, H_2O < 0.5 ppm) or under nitrogen atmosphere using conventional Schlenk techniques. Glassware was dried either overnight in a 120 °C oven or under vacuum using a *Steinel* heat gun. Solids were added to reaction mixtures in a nitrogen countercurrent. The addition of liquid reagents and solvents was performed by using disposable plastic syringes with disposable cannulas. For reactions performed at temperatures other than room temperature, the temperature of the surrounding oil bath, ice bath, or aluminum heating block is given as a reference.

Reagents and Solvents

MeCN, CH_2Cl_2, THF, 1,2-dichloroethane (DCE), 1,2-$Cl_2C_6H_4$, ClC_6H_5, toluene, and *n*-hexane were heated either over sodium using benzophenone as an indicator or over CaH_2, distilled under nitrogen atmosphere and stored over 4Å molecular sieves. DMF was ordered from Thermo Fisher Scientific (99.8%, extra dry, over molecular sieves, AcroSeal®) and used as received. All solvents used inside a glovebox were degassed using three freeze-pump-thaw cycles. For reactions which did not require Schlenk conditions, extractions, filtrations or flash column chromatography, technical grade solvents were distilled prior to use. Reagents were obtained from commercial suppliers and used as received. Sodium *tert*-butoxide, lithium-, sodium-, and potassium carbonate were dried overnight under vacuum ($1 \cdot 10^{-2}$ mbar) at 80 °C prior to use. All isatoic anhydrides were dried overnight under vacuum ($1 \cdot 10^{-2}$ mbar) at room temperature prior to use. All *N*-aryl-*N′*-trimethylsilyldiazenes were synthesized according to literature procedures.[S1]

Chromatography

Thin-layer chromatography was performed on Macherey-Nagel Alugram®Xtra SIL G/UV254 aluminum-backed TLC plates pre-coated with silica gel 60 with a layer thickness of 200 µm. Normal-phase column chromatography was performed on a Biotage Isolera OneTM system equipped with an Advian 30SIHP column using a mixture of cyclohexane and ethyl acetate as the eluent.

Nuclear Magnetic Resonance (NMR) Spectroscopy

^1H and ^{13}C NMR spectra were recorded on Bruker AV 400, AV 500, or AV 700 instruments using $CDCl_3$ or DMSO-d_6 as the deuterated solvent. The ^1H and ^{13}C chemical shifts are reported in parts per million (ppm) and are referenced to the residual protic solvent signal ($CHCl_3$: δ/ppm = 7.26, DMSO-d_5: δ/ppm = 2.50) or to the ^{13}C signal of the deuterated solvent ($CDCl_3$: δ/ppm = 77.16; DMSO-d_6: δ/ppm = 39.52). The data is reported as follows: Chemical shift, multiplicity (s = singlet, d = doublet, t = triplet, q = quartet, m = multiplet, m_c =

centrosymmetric multiplet, or any combinations thereof), coupling constants (Hz), and integration. The specified multiplicities are phenomenological and may not correspond to the theoretically expected multiplicities.

Gas-Liquid-Chromatography

Gas-Liquid-Chromatography (GLC) analyses were performed on an Agilent Technologies GC 8860 gas chromatograph equipped with an Agilent Technologies J&W HP-5 capillary column (length: 30 m × inner diameter: 0.32 mm, film thickness: 0.25 µm). Measurements were performed using the following protocol: Carrier gas: N_2; injector temperature: 250 °C, detector temperature: 275 °C; flow rate isobaric 11 psi; temperature program: start temperature 40 °C, heating rate 10°C/min, final temperature: 280 °C for 10 min.

Mass Spectrometry

High resolution mass spectrometry (HRMS) measurements were performed by the analytical facility of the Institut für Chemie, Technische Universität Berlin. The spectra were recorded on a Thermo Scientific LTQ Orbitrap XL device using ESI or APCI techniques for ionization. For compound characterization, the molecular ion peak is reported.

Liquid Chromatography–Mass Spectrometry

Liquid Chromatography–Mass Spectrometry (LC–MS) measurements were performed by the analytical facility of the Institut für Chemie, Technische Universität Berlin. The chromatograms were recorded on an Agilent Technologies 1200 analytical HPLC equipped with a Grace Grom-Sil-120-ODS-4-HE column connected to a Thermo Fisher Scientific LTQ Orbitrap XL mass spectrometer. A mixture of MeCN (+0.1% HCO_2H) and H_2O (+0.1% HCO_2H) was used as the mobile phase. The following gradient was used: 0–10 min, MeCN : H_2O = 20 : 80 → 100 : 0; 10–12 min, MeCN : H_2O = 0 : 100; 12–18 min, MeCN : H_2O = 20 : 80; flow: 0.3 mL/min.

Melting Points

Melting points (M.p.) were recorded on a Stuart SMP 20 device and are not corrected. The melting points of 2-azosubstituted benzoxazine-4-ones could not be measured as these compounds decompose at temperatures above 140 °C under air atmosphere.

Infrared (IR) Spectroscopy

IR spectra were recorded on an Agilent Technologies Cary 630 FT-IR Fourier transform infrared spectrometer equipped with a diamond ATR unit. Selected absorption maxima are reported in wavenumbers (cm^{-1}).

2 General Procedures

2.1 General Procedure for the Synthesis of Azo-Substituted Benzoxazine-4-ones using Sodium Carbonate as the Base in the Cyclization Step (GP 1)

In an argon-filled glovebox, an oven-dried 1.5 mL screw-capped vial equipped with a magnetic stirring bar was charged with sodium carbonate (31.8 mg, 0.300 mmol, 1.00 equiv) and the corresponding isatoic anhydride (0.300 mmol, 1.00 equiv). In a second vial, the corresponding silyldiazene (0.510 mmol, 1.70 equiv) was dissolved in the indicated solvent (0.3 mL), and the resulting blue mixture was added to the reaction vial. The parent vial was rinsed with the indicated solvent (0.3 mL), and the reaction was stirred for 16 h at the indicated temperature. MeOH (1.0 mL) was added to the resulting orange mixture, the solvent was removed under reduced pressure, and the resulting azocarboxamide was dried for 12 h under high vaccum ($1 \cdot 10^{-2}$ mbar). The crude product was used in the subsequent cyclization step without further purification.

A 10 mL vial equipped with a magnetic stirring bar and a rubber septum was charged with the azocarboxamide, Mukaiyama reagent (115 mg, 0.450 mmol, 1.50 equiv), and sodium carbonate (31.8 mg, 0.300 mmol, 1.00 equiv). The vial was subjected to 3 vacuum-nitrogen cycles, CH_2Cl_2 (2.4 mL) and MeCN (1.2 mL) were added, and the reaction mixture was stirred for 48 h at 25 °C. The solvent was removed under reduced pressure, CH_2Cl_2 (10 mL) was added, and the crude product was filtered through a pad of silica. The solvent was removed under reduced pressure, and the product was purified by automated normal-phase column chromatography.

2.2 General Procedure for the Synthesis of Azo-Substituted Benzoxazine-4-ones using Triethylamine as the Base in the Cyclization Step (GP 2)

In an argon-filled glovebox, an oven-dried 1.5 mL screw-capped vial equipped with a magnetic stirring bar was charged with sodium carbonate (31.8 mg, 0.300 mmol, 1.00 equiv) and the corresponding isatoic anhydride (0.300 mmol, 1.00 equiv). In a second vial, the corresponding silyldiazene (0.510 mmol, 1.70 equiv) was dissolved in the indicated solvent (0.3 mL), and the resulting blue mixture was added to the reaction vial. The parent vial was rinsed with the indicated solvent (0.3 mL), and the reaction was stirred for 16 h at the indicated temperature. MeOH (1.0 mL), EtOAc (30 mL), and HCl (1M, 30 mL) were added to the resulting orange mixture, the phases were separated and the aqueous phase was extracted with EtOAc (2 × 30 mL). The combined organic phases were dried over Mg_2SO_4, and the solvent was removed

under reduced pressure. The resulting azocarboxamide was dried for 6 h under high vaccum ($1 \cdot 10^{-2}$ mbar) and was used in the subsequent cyclization step without further purification.

A 10 mL vial equipped with a magnetic stirring bar and a rubber septum was charged with the azocarboxamide and Mukaiyama reagent (115 mg, 0.450 mmol, 1.50 equiv). The vial was subjected to 3 vacuum-nitrogen cycles, CH_2Cl_2 (2.4 mL), MeCN (1.2 mL), and Et_3N (85 µL, 0.610 mmol, 2.03 equiv) were added subsequently, and the reaction mixture was stirred for 48 h at 25 °C. The solvent was removed under reduced pressure, and the crude product was dried under high vacuum ($1 \cdot 10^{-2}$ mbar) for 30 min. CH_2Cl_2 (10 mL) was added, and the resulting suspension was filtered through a pad of silica. The solvent was removed under reduced pressure, and the product was purified by automated normal-phase column chromatography.

2.3 Optimization of the Ring Opening Reaction

In an argon-filled glovebox, an oven-dried 1.5 mL screw-capped vial equipped with a magnetic stirring bar was charged with the indicated base and isatoic anhydride (16.3 mg, 0.100 mmol, 1.00 equiv). In a second vial, the indicated amount of *N*-phenyl-*N'*-trimethylsilyldiazene was dissolved in the indicated solvent (0.1 mL) and the resulting blue mixture was added to the reaction vial. The parent vial was rinsed with the indicated solvent (0.1 mL) and the reaction mixture was stirred for 16 h at the indicated temperature. The vial was removed from the glovebox, and the reaction mixture was quenched with MeOH (10 mL). 2 mL of the resulting solution was transferred into a 10 mL round-bottom flask, the solvent was removed under reduced pressure, and 1 mL of a freshly prepared stock solution of 1,3,5-trimethoxybenzene (16.8 mg, 0.1 mmol) in *n*-pentane (5 mL) was added. After evaporation of the solvent, the mixture was dissolved in DMSO-d_6 and analyzed by quantitative NMR.

Table S1: Optimization of the Ring Opening of Isatoic Anhydrides.

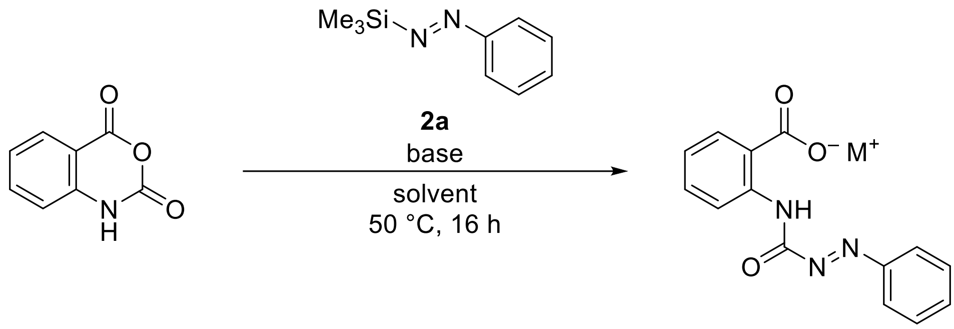

1a **3aa**

entry	base (equiv.)	solvent	**2a** (equiv.)	yield (%)[a]
1	$NaOSiMe_3$	1,2-$Cl_2C_6H_4$	2.0	0
2	Na**O*t*Bu** (1.0)	1,2-$Cl_2C_6H_4$	2.0	0
3	Na_2**CO_3** (1.0)	1,2-$Cl_2C_6H_4$	2.0	87
4	**Li_2**CO_3 (1.0)	1,2-$Cl_2C_6H_4$	2.0	70
5	**K_2**CO_3 (1.0)	1,2-$Cl_2C_6H_4$	2.0	71
6	**Cs_2**CO_3 (1.0)	1,2-$Cl_2C_6H_4$	2.0	56
7	Na_2CO_3 (**0.5**)	1,2-$Cl_2C_6H_4$	2.0	64
8	Na_2CO_3 (**0.2**)	1,2-$Cl_2C_6H_4$	2.0	52
9	Na_2CO_3 (1.0)	**toluene**	2.0	79
10	Na_2CO_3 (1.0)	**ClC_6H_5**	2.0	94
11	Na_2CO_3 (1.0)	**DCE**	2.0	61
12	Na_2CO_3 (1.0)	***n*-hexane**	2.0	83
13	Na_2CO_3 (1.0)	**THF**	2.0	70
14	Na_2CO_3 (1.0)	**DMF**	2.0	0
15	Na_2CO_3 (1.0)	ClC_6H_5	**1.7**	91
16	Na_2CO_3 (1.0)	ClC_6H_5	**1.4**	63
17	**---**	ClC_6H_5	1.7	0

All reactions were performed on a 0.1 mmol scale. [a] Determined by quantitative NMR using 1,3,5-trimethoxybenzene as internal standard.

2.4 Optimization of the Cyclization Reaction

A 1.5 mL screw-capped vial equipped with a magnetic stirring bar, was charged with (*E*)-2-(phenyldiazenecarboxamido)benzoic acid (13.5 mg, 50.1 µmol, 1.00 equiv), the indicated coupling reagent, the indicated base (if solid), and a known amount of docosane. 0.6 mL of the indicated solvent or solvent mixture (0.6 mL) and the indicated base (if liquid) were added, and the reaction mixture was stirred for the indicated time at 25 °C. An aliquot of the reaction mixture was taken and analyzed by GLC-analysis.

Table S2: Optimization of the Cyclization of Diazenecarboxamidobenzoates.

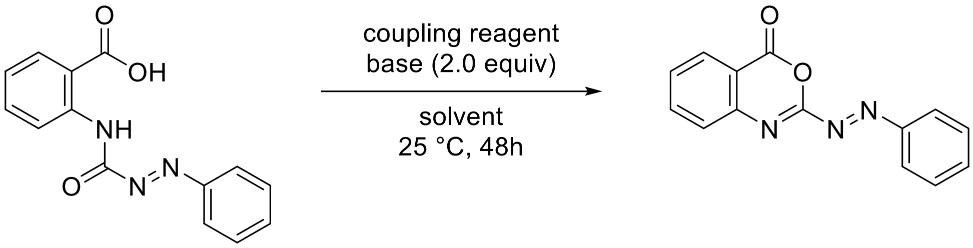

3aa **5aa**

entry	coupling reagent (equiv.)	solvent	base	yield (%)[a]
1	**DCC** (1.2) + **DMAP** (0.1)	CH_2Cl_2/MeCN = 2 : 1	Na_2CO_3	94
2	**CDI** (1.2)	CH_2Cl_2/MeCN = 2 : 1	Na_2CO_3	83
3	**$COCl_2$** (1.2)	CH_2Cl_2/MeCN = 2 : 1	Na_2CO_3	0
4	**PhCOCl** (1.2)	CH_2Cl_2/MeCN = 2 : 1	Na_2CO_3	93
5	**Mukaiyama reagent** (1.2)	CH_2Cl_2/MeCN = 2 : 1	Na_2CO_3	95
6	Mukaiyama reagent (**1.5**)	CH_2Cl_2/MeCN = 2 : 1	Na_2CO_3	98
7	Mukaiyama reagent (1.5)	**MeCN**	Na_2CO_3	89
8	Mukaiyama reagent (1.5)	CH_2Cl_2/MeCN = 2 : 1	**Et_3N**	80

All reactions were performed on a 0.05 mmol scale using 0.6 mL of solvent. [a] Determined by calibrated GLC-analysis using docosane as internal standard.

3 Experimental Details for the Synthesis of (*E*)-2-(Phenyldiazenecarboxamido)benzoic Acid (3aa·H)

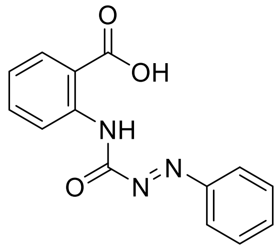

3aa·H
$C_{14}H_{11}N_3O_3$
M = 269.26 g/mol

In an argon-filled glovebox, an oven-dried 10 mL vial equipped with a magnetic stirring bar and a rubber septum was charged with potassium carbonate (27.6 mg, 0.200 mmol, 1.00 equiv) and isatoic anhydride (**1a**, 32.6 mg, 0.200 mmol, 1.00 equiv). In a second vial *N*-phenyl-*N'*-trimethylsilyldiazene (**2a**, 71.3 mg, 0.400 mmol, 2.00 equiv) was dissolved in 1,2-$Cl_2C_6H_4$ (0.2 mL) and the resulting blue mixture was added to the reaction vial. The parent vial was rinsed with 1,2-$Cl_2C_6H_4$ (0.2 mL), and the reaction was stirred for 16 h at 50 °C. The resulting orange mixture was transferred into a 50 mL round bottom flask, MeOH (10 mL) was added, and the reaction mixture was concentrated under reduced pressure. MTBE (20 mL) was added, and the resulting suspension was filtered. The filter cake was washed with MTBE (10 mL) and dissolved in MeOH (10 mL) again. The crude product was concentrated and precipitated 2 additional times before EtOAc (150 mL) and HCl (1M, 150 mL) were added. The phases were separated, and the organic phase filtered through a pad of $MgSO_4$. The solvent was removed under reduced pressure and (*E*)-2-(phenyl-diazenecarboxamido)benzoic acid (**3aa**·H, 33.5 mg, 0.124 mmol, 62%) was afforded as an orange solid.

M.p. = 142 °C (EtOAc). **^{1}H NMR** (500 MHz, DMSO-d_6): δ/ppm =13.70 (br s, 1H), 12.26 (br s, 1H), 8.59 (d, *J* = 8.4 Hz, 1H), 8.08 (d, *J* = 7.8 Hz, 1H), 7.97 (d, *J* = 7.8 Hz, 2H), 7.79–7.63 (m, 4H), 7.29 (t, *J* = 7.7 Hz, 1H). **^{13}C{^{1}H} NMR** (101 MHz, DMSO-d_6): δ/ppm = 169.4, 157.8, 150.6, 139.4, 134.4 (2C), 131.5, 129.9 (2C), 124.0, 123.6 (2C), 119.4, 117.6. **HRMS** (APCI) m/z: $[M + H]^+$ Calcd for $C_{14}H_{12}N_3O_3$ 270.0873; Found: 270.0871. **IR** (ATR) ṽ/cm^{-1} = 3438, 3203, 2920, 2850, 2653, 2441, 2328, 2237, 2109, 1728, 1666, 1580, 1521, 1446, 1408, 1310, 1264, 1164, 1087, 970, 892, 751, 678.

4 Experimental Details for the Synthesis of Azo-Substituted Benzoxazinones

(*E*)-2-(phenyldiazenyl)-4*H*-benzo[d][1,3]oxazin-4-one (5aa)

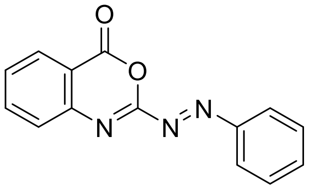

5aa
$C_{14}H_9N_3O_2$
M = 251.25 g/mol

Prepared according to **GP 1** from isatoic anhydride (**1a**, 48.9 mg, 0.300 mmol, 1.00 equiv) and (*E*)-1-phenyl-2-(trimethylsilyl)diazene (**2a**, 90.9 mg, 0.510 mmol, 1.70 equiv). The ring-opening reaction was performed in C_6H_5Cl at 50 °C. Purification by automated flash column chromatography on silica gel (cyclohexane : EtOAc = 1 : 0 → 9 : 1) afforded the title compound (**5aa**, 52.7 mg, 0.210 mmol, 70%) as an orange solid.

M.p. = decomposed. **R_f** = 0.13 (cyclohexane : ethyl acetate = 9 : 1). **^{1}H NMR** (500 MHz, $CDCl_3$): δ/ppm = 8.31 (d, *J* = 7.8 Hz, 1H), 8.13 (d, *J* = 7.6 Hz, 2H), 7.96–7.85 (m, 2H), 7.69–7.61 (m, 2H), 7.58 (t, *J* = 7.1 Hz, 2H). **^{13}C{^{1}H} NMR** (126 MHz, $CDCl_3$): δ/ppm = 158.6, 156.4, 152.5, 146.8, 137.2, 134.8, 130.1, 129.6 (2C), 129.4, 128.7, 124.8 (2C), 117.0. **HRMS** (APCI) m/z: $[M + H]^+$ Calcd for $C_{14}H_{10}N_3O_2$ 252.0768; Found: 252.0768. **IR** (ATR) $\tilde{\nu}$/cm^{-1} = 3530, 3205, 3061, 2785, 2292, 2139, 2126, 1915, 1767, 1600, 1460, 1429, 1307, 1260, 1215, 1186, 1147, 1037, 998, 891, 777, 706, 676.

(*E*)-5-methoxy-2-(phenyldiazenyl)-4*H*-benzo[d][1,3]oxazin-4-one (5ba)

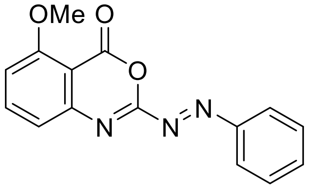

5ba
$C_{15}H_{11}N_3O_3$
M = 281.27 g/mol

Prepared according to **GP 1** from 5-methoxy-2*H*-benzo[d][1,3]oxazine-2,4(1H)-dione (**1b**, 54.3 mg, 0.281 mmol, 1.00 equiv) and (*E*)-1-phenyl-2-(trimethylsilyl)diazene (**2a**, 85.2 mg, 0.478 mmol, 1.70 equiv). The ring-opening reaction was performed in C_6H_5Cl at 50 °C. Purification by automated flash column chromatography on silica gel (cyclohexane : EtOAc

= 1 : 0 → 8 : 2) afforded the title compound (**5ba**, 39.1 mg, 0.139 mmol, 49%) as an orange solid.

M.p. = decomposed. **R$_f$** = 0.15 (cyclohexane : ethyl acetate = 9 : 1). **^{1}H NMR** (500 MHz, $CDCl_3$): δ/ppm = 8.12 (d, *J* = 8.1 Hz, 2H), 7.81 (t, *J* = 8.2 Hz, 1H), 7.63 (t, *J* = 7.5 Hz, 1H), 7.57 (t, *J* = 7.7 Hz, 2H), 7.47 (d, *J* = 8.1 Hz, 1H), 7.09 (d, *J* = 8.4 Hz, 1H), 4.06 (s, 3H). **^{13}C{^{1}H} NMR** (126 MHz, $CDCl_3$): δ/ppm = 161.6, 157.0, 155.1, 152.6, 149.1, 137.6, 134.7, 129.6 (2C), 124.8 (2C), 120.6, 111.7, 105.7, 56.8. **HRMS** (APCI) m/z: $[M + H]^+$ Calcd for $C_{15}H_{12}N_3O_3$ 282.0873; Found: 282.0873. **IR** (ATR) ṽ/cm^{-1} = 3185, 3064, 2939, 2781, 2340, 2111, 2076, 1867, 1775, 1593, 1477, 1427, 1309, 1241, 1185, 1147, 1077, 967, 895, 808, 768, 676.

(*E*)-5-chloro-2-(phenyldiazenyl)-4*H*-benzo[d][1,3]oxazin-4-one (5ca)

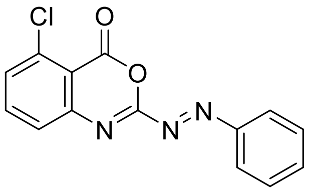

5ca
$C_{14}H_8ClN_3O_2$
M = 285.69 g/mol

M.p. = decomposed. Prepared according to **GP 2** from 5-chloro-2*H*-benzo[d][1,3]oxazine-2,4(1H)-dione (**1c**, 59.3 mg, 0.300 mmol, 1.00 equiv) and (*E*)-1-phenyl-2-(trimethylsilyl)diazene (**2a**, 91.0 mg, 0.510 mmol, 1.70 equiv). The ring-opening reaction was performed in *n*-hexane at room temperature. Purification by automated flash column chromatography on silica gel (cyclohexane : EtOAc = 1 : 0 → 9 : 1) afforded the title compound (**5ca**, 28.0 mg, 0.0980 mmol, 33%) as an orange solid.

R$_f$ = 0.11 (cyclohexane : ethyl acetate = 9 : 1). **^{1}H NMR** (400 MHz, $CDCl_3$): δ/ppm = 8.12 (d, *J* = 7.6 Hz, 2H), 7.81 (dd, *J* = 8.1, 1.6 Hz, 1H), 7.77 (t, *J* = 7.7 Hz, 1H), 7.65–7.62 (m, 2H), 7.58 (t, *J* = 7.9 Hz, 2H). **^{13}C{^{1}H} NMR** (100 MHz, $CDCl_3$): δ/ppm = 156.7, 155.2, 152.5, 149.1, 137.0, 136.5, 135.1, 132.5, 129.7 (2C), 127.6, 124.9 (2C), 114.7. **HRMS** (APCI) m/z: $[M + H]^+$ Calcd for $C_{14}H_9ClN_3O_2$ 286.0378; Found: 286.0875. **IR** (ATR) ṽ/cm^{-1} = 3555, 3077, 2922, 2599, 2340, 2109, 1906, 1777, 1619, 1588, 1453, 1419, 1300, 1245, 1188, 1144, 979, 936, 894, 811, 770, 735, 671.

(*E*)-6-methoxy-2-(phenyldiazenyl)-4*H*-benzo[d][1,3]oxazin-4-one (5da)

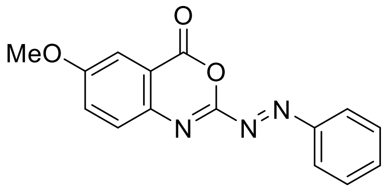

5da

$C_{15}H_{11}N_3O_3$

M = 281.27 g/mol

Prepared according to **GP 1** from 6-methoxy-2*H*-benzo[d][1,3]oxazine-2,4(1H)-dione (**1d**, 58.0 mg, 0.300 mmol, 1.00 equiv) and (*E*)-1-phenyl-2-(trimethylsilyl)diazene (**2a**, 91.0 mg, 0.510 mmol, 1.70 equiv). The ring-opening reaction was performed in C_6H_5Cl at 50 °C. Purification by automated flash column chromatography on silica gel (cyclohexane : EtOAc = 1 : 0 → 8 : 2) afforded the title compound (**5da**, 56.3 mg, 0.200 mmol, 67%) as an orange solid.

M.p. = decomposed. $\mathbf{R_f}$ = 0.15 (cyclohexane : ethyl acetate = 8 : 2). ^{1}**H NMR** (500 MHz, $CDCl_3$): δ/ppm = 8.11 (d, *J* = 7.9 Hz, 2H), 7.84 (d, *J* = 8.88 Hz, 1H), 7.69 (d, *J* = 2.7 Hz, 1H), 7.62 (t, *J* = 7.2 Hz, 1H), 7.57 (t, *J* = 7.2 Hz, 2H), 7.47 (dd, *J* = 8.9, 3.0 Hz, 1H), 3.96 (s, 3H). ^{13}**C{**^{1}**H} NMR** (126 MHz, $CDCl_3$): δ/ppm = 160.9, 158.8, 155.0, 152.6, 140.9, 134.5, 130.3, 129.6 (2C), 126.3, 124.7 (2C), 118.1, 109.9, 56.3. **HRMS** (APCI) m/z: $[M + H]^+$ Calcd for $C_{15}H_{12}N_3O_3$ 282.0873; Found: 282.0873. **IR** (ATR) ṽ/cm^{-1} = 3485, 2937, 2625, 2329, 2116, 2001, 1910, 1747, 1592, 1491, 1447, 1350, 1272, 1240, 1142, 1013, 928, 833, 775, 732, 711, 675.

(*E*)-6-chloro-2-(phenyldiazenyl)-4*H*-benzo[d][1,3]oxazin-4-one (5ea)

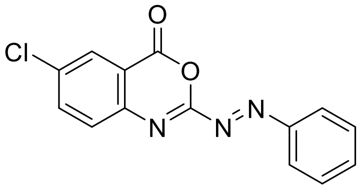

5ea

$C_{14}H_8ClN_3O_2$

M = 285.69 g/mol

Prepared according to **GP 2** from 6-chloro-2*H*-benzo[d][1,3]oxazine-2,4(1H)-dione (**1e**, 59.1 mg, 0.300 mmol, 1.00 equiv) and (*E*)-1-phenyl-2-(trimethylsilyl)diazene (**2a**, 91.0 mg, 0.510 mmol, 1.70 equiv). The ring-opening reaction was performed in *n*-hexane at room temperature. Purification by automated flash column chromatography on silica gel

(cyclohexane : EtOAc = 1 : 0 → 9 : 1) afforded the title compound (**5ea**, 55.9 mg, 0.196 mmol, 65%) as an orange solid.

M.p. = decomposed. **R$_f$** = 0.13 (cyclohexane : ethyl acetate = 9 : 1). **^{1}H NMR** (500 MHz, $CDCl_3$): δ/ppm = 8.24 (s, 1H), 8.10 (d, *J* = 7.9 Hz, 2H), 7.83 (s, 2H), 7.63 (t, *J* = 7.5 Hz, 1H), 7.57 (t, *J* = 7.8 Hz, 2H). **^{13}C{^{1}H} NMR** (100 MHz, $CDCl_3$): δ/ppm = 157.5, 156.3, 152.4, 145.2, 137.5, 135.9, 135.1, 130.1, 129.6 (2C), 128.7, 124.8 (2C), 118.0. **HRMS** (APCI) m/z: $[M + H]^+$ Calcd for $C_{14}H_9ClN_3O_2$ 286.0378; Found: 286.0375. **IR** (ATR) ṽ/cm^{-1} = 3061, 2921, 2326, 2115, 1995, 1902, 1769, 1592, 1464, 1422, 1308, 1250, 1181, 1147, 1131, 1028, 977, 889, 842, 804, 772, 711, 672.

(*E*)-6-methyl-2-(phenyldiazenyl)-4*H*-benzo[d][1,3]oxazin-4-one (5fa)

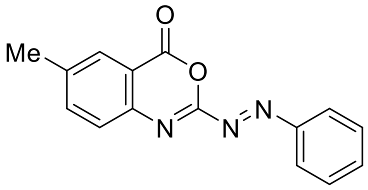

5fa
$C_{15}H_{11}N_3O_2$
M = 265.27 g/mol

Prepared according to **GP 1** from 6-methyl-2*H*-benzo[d][1,3]oxazine-2,4(1H)-dione (**1f**, 53.2 mg, 0.300 mmol, 1.00 equiv) and (*E*)-1-phenyl-2-(trimethylsilyl)diazene (**2a**, 91.0 mg, 0.510 mmol, 1.70 equiv). The ring-opening reaction was performed in C_6H_5Cl at 50 °C. Purification by automated flash column chromatography on silica gel (cyclohexane : EtOAc = 1 : 0 → 9 : 1) afforded the title compound (**5fa**, 56.7 mg, 0.214 mmol, 71%) as an orange solid.

M.p. = decomposed. **R$_f$** = 0.14 (cyclohexane : ethyl acetate = 8 : 2). **^{1}H NMR** (500 MHz, $CDCl_3$): δ/ppm = 8.16–8.02 (m, 3H), 7.79 (d, *J* = 8.4 Hz, 1H), 7.70 (d, *J* = 8.2 Hz, 1H), 7.61 (t, *J* = 7.2 Hz, 1H), 7.56 (t, *J* = 7.7 Hz, 2H), 2.52 (s, 3H). **^{13}C{^{1}H} NMR** (126 MHz, $CDCl_3$): δ/ppm = 158.7, 155.9, 152.5, 144.6, 140.9, 138.3, 134.6, 129.6, 129.0, 128.5, 124.7, 116.7, 21.6. **HRMS** (ESI) m/z: $[M + H]^+$ Calcd for $C_{15}H_{12}N_3O_2$ 266.0924; Found: 266.0920. **IR** (ATR) ṽ/cm^{-1} = 3497, 3196, 3066, 2923, 2857, 2746, 2639, 2340, 2114, 1998, 1903, 1752, 1599, 1464, 1434, 1313, 1269, 1222, 1191, 1144, 1035, 983, 897, 830, 773, 709, 676.

(*E*)-7-methoxy-2-(phenyldiazenyl)-4*H*-benzo[d][1,3]oxazin-4-one (5ga)

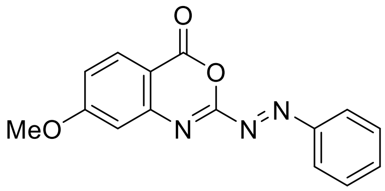

5ga
$C_{15}H_{11}N_3O_3$
M = 281.27 g/mol

Prepared according to **GP 2** from 7-methoxy-2*H*-benzo[d][1,3]oxazine-2,4(1H)-dione (**1g**, 58.0 mg, 0.300 mmol, 1.00 equiv) and (*E*)-1-phenyl-2-(trimethylsilyl)diazene (**2a**, 90.9 mg, 0.510 mmol, 1.70 equiv). The ring-opening reaction was performed in C_6H_5Cl at 50 °C. Purification by automated flash column chromatography on silica gel (cyclohexane : EtOAc = 1 : 0 → 8 : 2) afforded the title compound (**5ga**, 32.1 mg, 0.114 mmol, 38%) as an orange solid.

M.p. = decomposed. **R$_f$** = 0.13 (cyclohexane : ethyl acetate = 9 : 1). **^{1}H NMR** (500 MHz, $CDCl_3$): δ/ppm = 8.19 (d, *J* = 8.8 Hz, 1H), 8.12 (d, *J* = 8.1 Hz, 2H), 7.63 (t, *J* = 7.4 Hz, 1H), 7.57 (t, *J* = 7.9 Hz, 2H), 7.31 (d, *J* = 2.3 Hz, 1H), 7.16 (dd, *J* = 8.9, 2.3 Hz, 1H), 3.96 (s, 3H). **^{13}C{^{1}H} NMR** (126 MHz, $CDCl_3$): δ/ppm = 166.8, 158.2, 157.3, 152.6, 149.3, 134.8, 130.9, 129.6 (2C), 124.8 (2C), 118.8, 110.6, 109.6, 56.2. **HRMS** (APCI) m/z: $[M + H]^+$ Calcd for $C_{15}H_{12}N_3O_3$ 282.0873; Found: 282.0869. **IR** (ATR) ṽ/cm^{-1} = 2920, 2642, 2329, 2123, 1997, 1919, 1753, 1598, 1463, 1426, 1353, 1279, 1185, 1147, 1109, 1026, 981, 853, 773, 714, 671.

(*E*)-7-chloro-2-(phenyldiazenyl)-4*H*-benzo[d][1,3]oxazin-4-one (5ha)

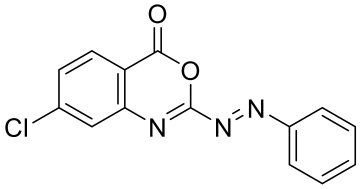

5ha
$C_{14}H_8ClN_3O_2$
M = 285.69 g/mol

Prepared according to **GP 2** from 7-chloro-2*H*-benzo[d][1,3]oxazine-2,4(1H)-dione (**1h**, 59.2 mg, 0.300 mmol, 1.00 equiv) and (*E*)-1-phenyl-2-(trimethylsilyl)diazene (**2a**, 91.0 mg, 0.510 mmol, 1.70 equiv). The ring-opening reaction was performed in *n*-hexane at room temperature. Purification by automated flash column chromatography on silica gel (cyclohexane : EtOAc = 1 : 0 → 9 : 1) afforded the title compound (**5ha**, 59.3 mg, 0.208 mmol, 69%) as an orange solid.

M.p. = decomposed. **R$_f$** = 0.25 (cyclohexane : ethyl acetate = 9 : 1). **^{1}H NMR** (500 MHz, $CDCl_3$): δ/ppm = 8.23 (d, *J* = 8.4 Hz, 1H), 8.12 (d, *J* = 8.0 Hz, 2H), 7.88 (d, *J* = 1.8 Hz, 1H), 7.65 (t, *J* = 7.2 Hz, 1H), 7.62–7.54 (m, 3H). **^{13}C{^{1}H} NMR** (126 MHz, $CDCl_3$): δ/ppm = 157.8, 157.3, 152.5, 147.9, 143.7, 135.1, 130.5, 130.5, 129.7 (2C), 128.3, 124.9 (2C), 115.3. **HRMS** (ESI) m/z: $[M + H]^+$ Calcd for $C_{14}H_9ClN_3O_2$ 286.0378; Found: 286.0375. **IR** (ATR) ṽ/cm^{-1} = 3626, 3168, 3069, 2920, 2850, 2323, 2112, 1997, 1901, 1766, 1587, 1457, 1419, 1310, 1246, 1178, 1139, 1076, 999, 922, 879, 838, 774, 712, 570.

(*E*)-7-fluoro-2-(phenyldiazenyl)-4*H*-benzo[d][1,3]oxazin-4-one (5ia)

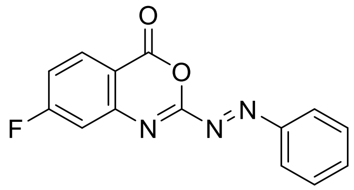

5ia
$C_{14}H_8FN_3O_2$
M = 269.24 g/mol

Prepared according to **GP 1** from 7-fluoro-2*H*-benzo[d][1,3]oxazine-2,4(1H)-dione (**1i**, 54.3 mg, 0.300 mmol, 1.00 equiv) and (*E*)-1-phenyl-2-(trimethylsilyl)diazene (**2a**, 91.0 mg, 0.510 mmol, 1.70 equiv). The ring-opening reaction was performed in *n*-hexane at room temperature. Purification by automated flash column chromatography on silica gel (cyclohexane : EtOAc = 1 : 0 → 9 : 1) afforded the title compound (**5ia**, 43.6 mg, 0.162 mmol, 54%) as an orange solid.

M.p. = decomposed. **R$_f$** = 0.20 (cyclohexane : ethyl acetate = 9 : 1). **^{1}H NMR** (500 MHz, $CDCl_3$): δ/ppm = 8.33 (dd, *J* = 8.5, 6.1 Hz, 1H), 8.12 (d, *J* = 7.6 Hz, 2H), 7.66 (t, *J* = 7.2 Hz, 1H), 7.63–7.50 (m, 3H), 7.35 (t, *J* = 8.1 Hz, 1H). **^{13}C{^{1}H} NMR** (101 MHz, $CDCl_3$): δ/ppm = 168.1 (d, *J* = 259.4 Hz), 157.7, 157.3, 152.5, 149.4 (d, *J* = 13.3 Hz), 135.2, 132.1 (d, *J* = 10.9 Hz), 129.7 (2C), 124.9 (2C), 118.4 (d, *J* = 23.5 Hz), 114.8 (d, *J* = 23.0 Hz), 113.5 (d, *J* = 2.8 Hz). **^{19}F NMR** (471 MHz, $CDCl_3$): δ/ppm = –97.7–(–97.5) (m, 1F). **HRMS** (ESI) m/z: $[M + H]^+$ Calcd for $C_{14}H_9FN_3O_2$ 270.0673; Found: 270.0673. **IR** (ATR) ṽ/cm^{-1} = 3088, 2919, 2325, 2217, 2123, 2100, 1997, 1925, 1908, 1772, 1599, 1478, 1418, 1328, 1281, 1217, 1185, 1141, 984, 872, 831, 772, 712, 668.

(*E*)-2-(*p*-tolyldiazenyl)-4*H*-benzo[d][1,3]oxazin-4-one (5ab)

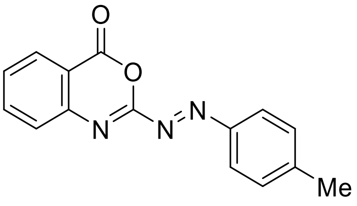

5ab
$C_{15}H_{11}N_3O_2$
M = 265.27 g/mol

Prepared according to **GP 1** from isatoic anhydride (**1a**, 48.9 mg, 0.300 mmol, 1.00 equiv) and (*E*)-1-(4-tolyl)-2-(trimethylsilyl)diazene (**2b**, 98.1 mg, 0.510 mmol, 1.70 equiv). The ring-opening reaction was performed in C_6H_5Cl at 50 °C. Purification by automated flash column chromatography on silica gel (cyclohexane : EtOAc = 1 : 0 → 9 : 1) afforded the title compound (**5ab**, 50.6 mg, 0.191 mmol, 64%) as an orange solid.

M.p. = decomposed. **R_f** = 0.15 (cyclohexane : ethyl acetate = 9 : 1). **^{1}H NMR** (500 MHz, $CDCl_3$): δ/ppm = 8.29 (d, *J* = 7.9 Hz, 1H), 8.02 (d, *J* = 8.3 Hz, 2H), 7.92–7.85 (m, 2H), 7.65–7.58 (m, 1H), 7.37 (d, *J* = 8.2 Hz, 2H), 2.46 (s, 3H). **^{13}C{^{1}H} NMR** (126 MHz, $CDCl_3$): δ/ppm = 158.7, 156.6, 150.9, 146.9, 146.4, 137.1, 130.3 (2C), 129.8, 129.3, 128.6, 124.9 (2C), 116.9, 22.0. **HRMS** (APCI) m/z: $[M + H]^+$ Calcd for $C_{15}H_{12}N_3O_2$ 266.0924; Found: 266.0922. **IR** (ATR) ṽ/cm^{-1} = 3508, 3033, 2921, 2851, 2614, 2341, 2282, 2116, 1917, 1757, 1595, 1432, 1296, 1259, 1220, 1184, 1143, 1034, 1000, 882, 821, 771, 681.

(*E*)-2-((4-(trifluoromethoxy)phenyl)diazenyl)-4*H*-benzo[d][1,3]oxazin-4-one (5ac)

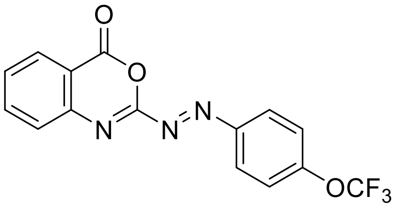

5ac
$C_{15}H_8F_3N_3O_3$
M = 335.24 g/mol

Prepared according to **GP 2** from isatoic anhydride (**1a**, 48.9 mg, 0.300 mmol, 1.00 equiv) and (*E*)-1-(4-(trifluoromethoxy)phenyl)-2-(trimethylsilyl)diazene (**2c**, 134 mg, 0.511 mmol, 1.70 equiv). The ring-opening reaction was performed in C_6H_5Cl at 50 °C. Purification by automated flash column chromatography on silica gel (cyclohexane : EtOAc = 1 : 0 → 9 : 1) afforded the title compound (**5ac**, 38.1 mg, 0.114 mmol, 38%) as an orange solid.

M.p. = decomposed. **R$_f$** = 0.13 (cyclohexane : ethyl acetate = 9 : 1). **^{1}H NMR** (500 MHz, $CDCl_3$): δ/ppm = 8.35–8.27 (m, 1H), 8.25–8.11 (m, 2H), 7.96–7.87 (m, 2H), 7.70–7.61 (m, 1H), 7.41 (d, *J* = 8.1 Hz, 2H). **^{13}C{^{1}H} NMR** (126 MHz, $CDCl_3$): δ/ppm = 158.4, 156.2, 153.7, 150.3, 146.7, 137.6–137.1 (m), 130.4 (d, *J* = 12.6 Hz), 129.5 (d, *J* = 7.5 Hz, 2C), 129.0–128.5 (m), 126.6 (d, *J* = 9.6 Hz), 121.3 (d, *J* = 10.7 Hz, 2C), 120.4 (q, *J* = 260.3 Hz), 116.9. **^{19}F NMR** (471 MHz, $CDCl_3$): δ/ppm = –57.6 (s, 3F). **HRMS** (APCI) m/z: $[M + H]^+$ Calcd for $C_{15}H_9F_3N_3O_3$ 336.0591; Found: 336.0589. **IR** (ATR) ṽ/cm^{-1} = 3527, 3192, 3068, 2925, 2784, 2419, 2323, 2117, 2096, 1992, 1924, 1764, 1588, 1466, 1413, 1245, 1139, 1035, 995, 900, 846, 775, 683.

(*E*)-2-((4-(trifluoromethyl)phenyl)diazenyl)-4*H*-benzo[d][1,3]oxazin-4-one (5ad)

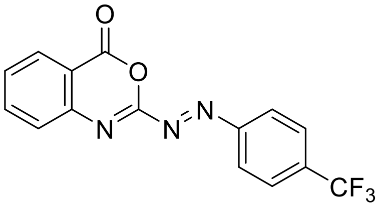

5ad
$C_{15}H_8F_3N_3O_2$
M = 319.06 g/mol

Prepared according to **GP 2** from isatoic anhydride (**1a**, 48.9 mg, 0.300 mmol, 1.00 equiv) and (*E*)-1-(4-(trifluoromethyl)phenyl)-2-(trimethylsilyl)diazene (**2d**, 120.5 mg, 0.510 mmol, 1.70 equiv). The ring-opening reaction was performed in C_6H_5Cl at 50 °C. Purification by automated flash column chromatography on silica gel (cyclohexane : EtOAc = 1 : 0 → 9 : 1) afforded the title compound (**5ad**, 34.2 mg, 0.107 mmol, 36%) as an orange solid.

M.p. = decomposed. **R$_f$** = 0.15 (cyclohexane : ethyl acetate = 9 : 1). **^{1}H NMR** (400 MHz, $CDCl_3$): δ/ppm = 8.33 (d, *J* = 7.7 Hz, 1H), 8.22 (d, *J* = 8.0 Hz, 2H), 7.99–7.90 (m, 2H), 7.86 (d, *J* = 8.0 Hz, 2H), 7.74–7.63 (m, 1H). **^{13}C{^{1}H} NMR** (126 MHz, $CDCl_3$): δ/ppm = 158.2, 156.0, 153.9, 146.5, 137.6–137.1 (m), 135.5 (q, *J* = 33 Hz), 130.6 (d, *J* = 26 Hz), 129.5 (d, *J* = 12 Hz), 129.1–128.6 (m), 126.9 (d, *J* = 25 Hz, 2C), 124.8 (d, *J* = 16 Hz, 2C), 123.6 (q, *J* = 273 Hz), 117.0. **^{19}F NMR** (471 MHz, $CDCl_3$): δ/ppm = –63.1 (s, 3F). **HRMS** (APCI) m/z: $[M + H]^+$ Calcd for $C_{15}H_9F_3N_3O_2$ 320.0641; Found: 320.0639. **IR** (ATR) ṽ/cm^{-1} = 3512, 2925, 2601, 2285, 2163, 2108, 2051, 1988, 1900, 1756, 1596, 1450, 1409, 1319, 1261, 1184, 1119, 1063, 1000, 900, 849, 771, 683.

(*E*)-2-((4-chlorophenyl)diazenyl)-4*H*-benzo[d][1,3]oxazin-4-one (5ae)

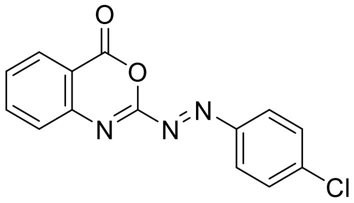

5ae
$C_{14}H_8ClN_3O_2$
M = 285.69 g/mol

Prepared according to **GP 2** from isatoic anhydride (**1a**, 48.9 mg, 0.300 mmol, 1.00 equiv) and methyl (*E*)-4-((trimethylsilyl)diazenyl)benzoate (**2e**, 120.5 mg, 0.510 mmol, 1.70 equiv). The ring-opening reaction was performed in C_6H_5Cl at 50 °C. Due to poor solubility of the intermediate azocarboxamide, the cyclization reaction was performed at lower concentration (MeCN: 2.5 mL, CH_2Cl_2: 5 mL) Purification by automated flash column chromatography on silica gel (cyclohexane : EtOAc = 1 : 0 → 6 : 4) afforded the title compound (**5ae**, 67.7 mg, 0.237 mmol, 79%) as an orange solid.

M.p. = decomposed. **R_f** = 0.13 (cyclohexane : ethyl acetate = 9 : 1). **^{1}H NMR** (400 MHz, $CDCl_3$): δ/ppm = 8.31 (d, *J* = 8.2 Hz, 1H), 8.08 (d, *J* = 8.8 Hz, 2H), 7.97–7.88 (m, 2H), 7.66 (ddd, *J* = 8.2, 6.3, 2.2 Hz, 1H), 7.56 (d, *J* = 8.8 Hz, 2H). **^{13}C{^{1}H} NMR** (101 MHz, $CDCl_3$): δ/ppm = 158.5, 156.2, 150.8, 146.7, 141.4, 137.3, 130.3, 130.1 (2C), 129.4, 128.7, 126.0 (2C), 116.9. **HRMS** (APCI) m/z: $[M + H]^+$ Calcd for $C_{14}H_9ClN_3O_2$ 286.0378; Found: 286.0372. **IR** (ATR) ṽ/cm^{-1} = 3092, 2921, 2227, 2127, 1944, 1914, 1892, 1760, 1595, 1473, 1440, 1399, 1295, 1260, 1215, 1182, 1144, 1087, 1036, 999, 900, 833, 770, 681.

5 Synthesis of (*E*)-2-(Phenyldiazenyl)-4*H*-benzo[d][1,3]oxazin-4-one (5aa) on a 1.0 mmol Scale

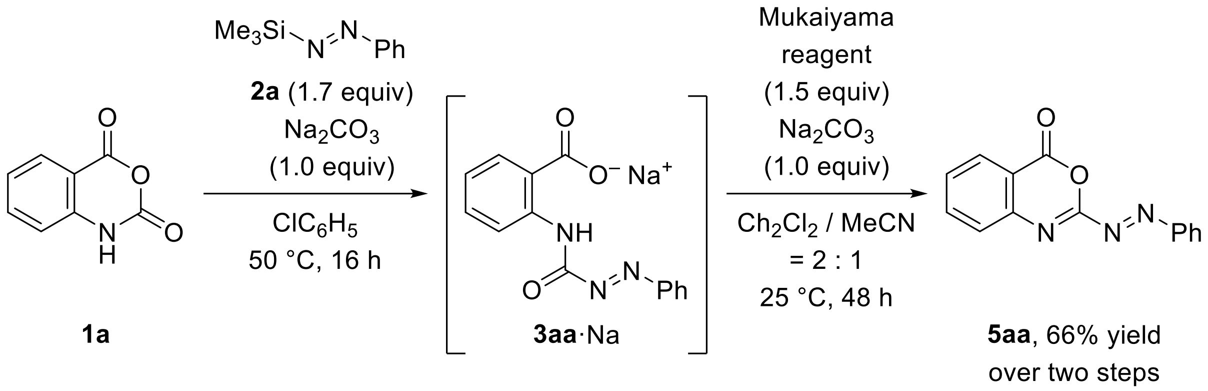

In a nitrogen-filled glovebox, an oven-dried 10 mL screw-capped vial equipped with a magnetic stirring bar, was charged with sodium carbonate (106 mg, 1.00 mmol, 1.00 equiv) and isatoic anhydride (**1a**, 163.1 mg, 1.00 mmol, 1.00 equiv). In a second vial, (*E*)-1-phenyl-2-(trimethylsilyl)diazene (**2a**, 303.1 mg, 1.70 mmol, 1.70 equiv) was dissolved in ClC_6H_5 (1.00 mL), and the resulting blue mixture was added to the reaction vial. The parent vial was rinsed with ClC_6H_5 (1.00 mL) and the reaction was stirred for 16 h at 50 °C. MeOH (10.0 mL) was added to the resulting orange mixture, the solvent was removed under reduced pressure, and the resulting azocarboxamide was dried for 12 hours under high vaccum ($1 \cdot 10^{-2}$ mbar). The crude product was used in the subsequent cyclization step without further purification.

A 25 mL round-bottom flask equipped with a magnetic stirring bar and a rubber septum was charged with the crude azocarboxamide **3aa**·Na, Mukaiyama reagent (383 mg, 1.50 mmol, 1.50 equiv), and sodium carbonate (106 mg, 1.00 mmol, 1.00 equiv). The flask was subjected to 3 nitrogen-vacuum cycles, and CH_2Cl_2 (8.0 mL) and MeCN (4.0 mL) were added. The reaction mixture was stirred for 48 h at 25 °C, the solvent was removed under reduced pressure, CH_2Cl_2 (30 mL) was added, and the crude product was filtered through a pad of silica. The solvent was removed under reduced pressure and the product was purified by automated normal-phase column chromatography (cyclohexane : EtOAc = 1 : 0 → 9 : 1). The product (**5aa**, 166 mg, 0.661 mmol, 66%) was received as an orange solid.

The spectroscopic data is in accordance with those previously reported in this document.

6 Experimental Details for the Synthesis of *N*-Methylisatoic Anhydride (9)

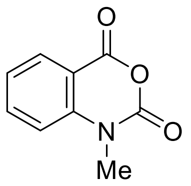

9

$C_9H_7NO_3$

M = 177.16 g/mol

A flame-dried 100 mL was charged with isatoic anhydride (**1a**, 3.02 g, 18.5 mmol, 1.00 equiv) and DMF (20 mL). K_2CO_3 (3.33 g, 24.1 mmol, 1.30 equiv) and MeI (1.5 mL, 3.42 g, 24.1 mmol, 1.30 equiv) were added in succession, and the reaction mixture was stirred for 17 h at room temperature. The reaction mixture was poured on water (80 mL), and the suspension was filtered through a fritted funnel. After purification by recrystallization (CH_2Cl_2 : MeOH), the product (**9**, 484 mg, 2.73 mmol, 15%) was received as a colorless solid.

^{1}H NMR (500 MHz, DMSO-d_6): δ/ppm = 8.01 (d, J = 7.8 Hz, 1H), 7.86 (t, J = 7.9 Hz, 1H), 7.45 (d, J = 8.5 Hz, 1H), 7.34 (t, J = 7.5 Hz, 1H), 3.47 (s, 3H). **^{13}C{^{1}H} NMR** (126 MHz, DMSO-d_6): δ/ppm = 159.0, 147.7, 142.2, 137.1, 129.3, 123.5, 114.8, 111.5, 31.6.

The spectroscopic data are in accordance with those reported in the literature.[S2]

7 Decomposition of *N*-Phenyl-*N′*-trimethylsilyldiazene 2a in MeOH

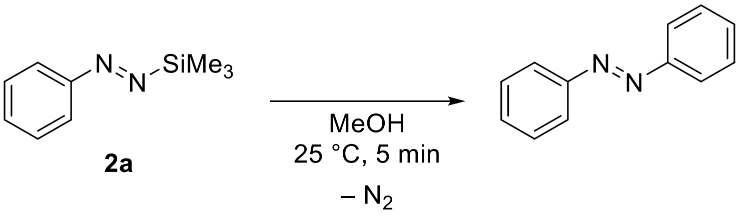

One drop of *N*-phenyl-*N′*-trimethylsilyldiazene **2a** was dissolved in MeOH (0.2 mL). After 30 s the solvent was removed under reduced pressure, and the resulting orange residue was analyzed by ^{1}H and $^{13}C\{^{1}H\}$ NMR spectroscopy.

Figure S1: ^{1}H NMR spectrum (500 MHz, $CDCl_3$) obtained for the decomposition of *N*-phenyl-*N′*-trimethylsilyldiazene (**2a**) in MeOH.

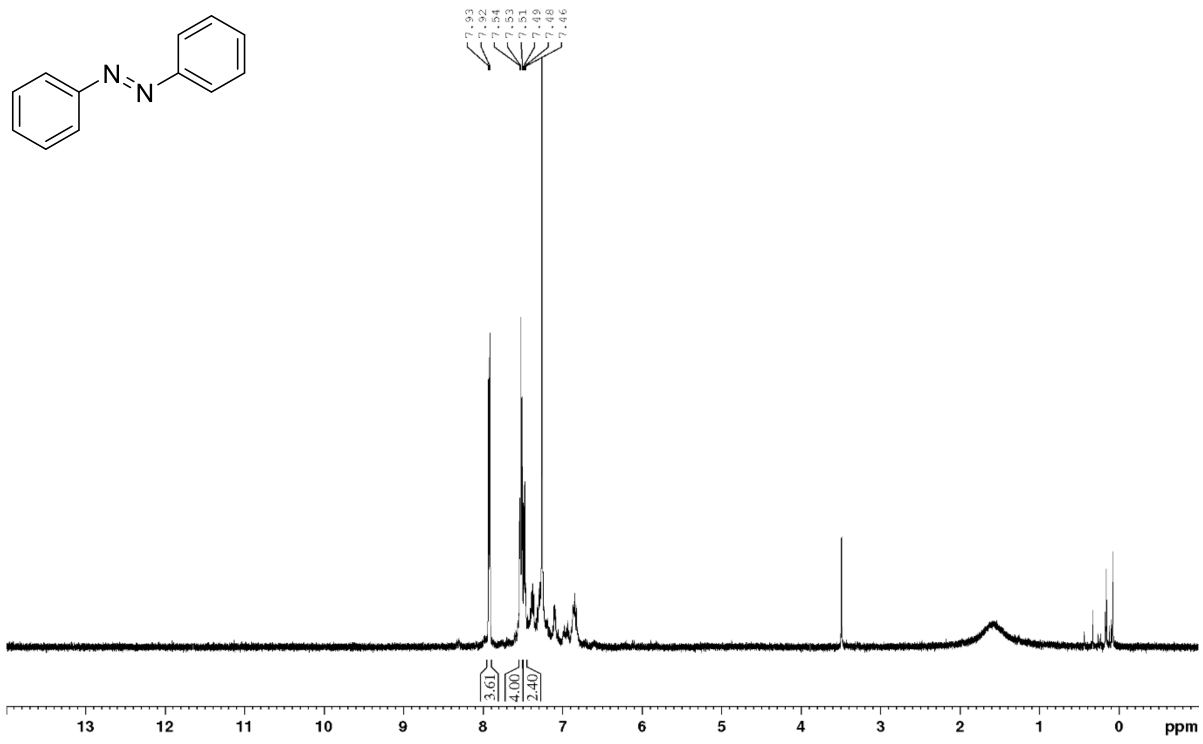

Figure S2: $^{13}C\{^1H\}$ NMR spectrum (101 MHz, $CDCl_3$) obtained for the decomposition of *N*-phenyl-*N'*-trimethylsilyldiazene (**2a**) in MeOH.

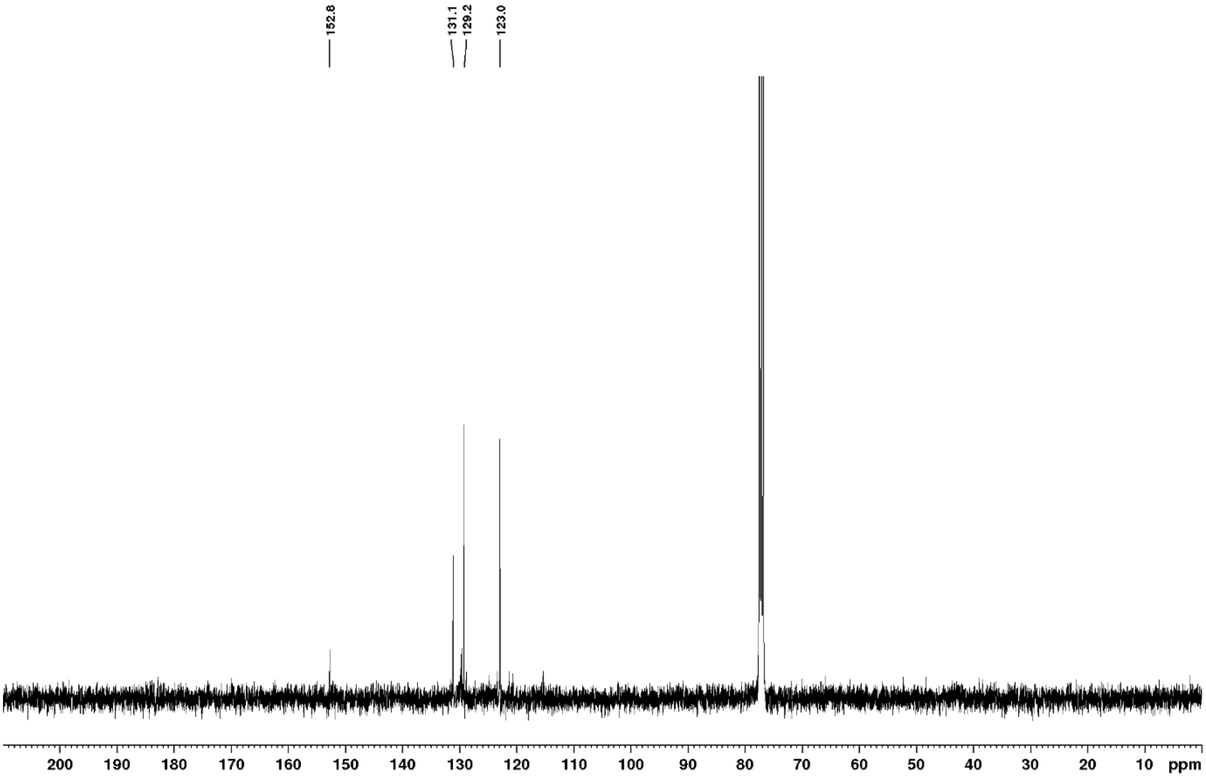

The NMR spectra are in accordance with those recorded for azobenzene in the literature.[S3]

8 Crystallographic Data

The data for the single-crystal structure determination was collected with an Agilent SuperNova diffractometer equipped with a CCD area Atlas detector and a mirror monochromator using Cu-K_α radiation (λ = 1.5418 Å). The following software packages were used: CrysAlisPro for data collection, cell refinement, and data reduction,[S4] SHELXS-2018 for structure solution,[S5] SHELXL-2018 for structure refinement,[S6] and Mercury for graphics.[S7] The single crystal of **5aa** was obtained through recrystallisation at room temperature using the vapor diffusion technique with EtOAc/*n*-pentane as solvents.

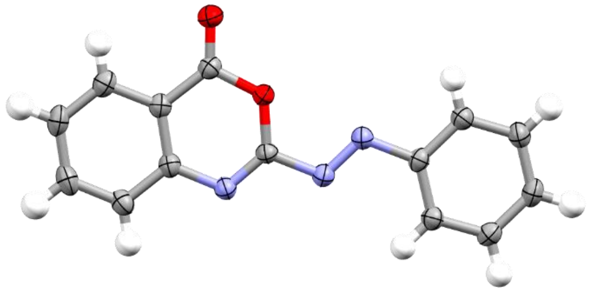

Figure S3: ORTEP view of the molecular structure of (*E*)-2-(phenyldiazenyl)-4*H*-benzo[d][1,3]oxazin-4-one (**5aa**) with thermal ellipsoids shown at the 50% probability level. Carbon atoms are colored in

gray, hydrogen atoms are colored in white, oxygen atoms are colored in red and nitrogen atoms are colored in blue. The structure can be found under CCDC 2553191.

Table S3: Crystal Data and Structure Refinement for (*E*)-2-(phenyldiazenyl)-4*H*-benzo[d] [1,3]oxazin-4-one (**5aa**).

Empirical formula	$C_{14}H_9N_3O_2$	
Formula weight	251.24	
Temperature	150.01(10) K	
Wavelength	1.54184 Å	
Crystal system	Orthorhombic	
Space group	Pbca (No. 61)	
Unit cell dimensions	a = 12.4239(4) Å	α = 90°
	b = 7.0771(2) Å	β = 90°
	c = 26.6625(9) Å	γ = 90°
Volume	2344.31(13) $Å^3$	
Z	8	
Density (calculated)	1.424 Mg/m^3	
Absorption coefficient	0.816 mm^{-1}	
F(000)	1040	
Crystal size	0.322 x 0.104 x 0.034 mm^3	
Theta range for data collection	3.315 to 72.576°	
Index ranges	–15<=h<=14, –8<=k<=7, –26<=l<=32	
Reflections collected	8286	
Independent reflections	2298 [R(int) = 0.0280]	
Completeness to theta = 67.684°	100.0 %	
Absorption correction	Semi-empirical from equivalents	
Max. and min. transmission	1.00000 and 0.30393	
Refinement method	Full-matrix least-squares on F^2	
Data / restraints / parameters	2298 / 0 / 172	
Goodness-of-fit on F^2	1.073	
Final R indices [I>2sigma(I)]	R1 = 0.0373, wR2 = 0.0988	
R indices (all data)	R1 = 0.0499, wR2 = 0.1088	
Extinction coefficient	n/a	
Largest diff. peak and hole	0.165 and -0.223 e.$Å^{-3}$	

9 UV/vis Spectrum of (*E*)-2-(Phenyldiazenyl)-4*H*-benzo[d][1,3]oxazin-4-one (5aa)

UV/vis Absorption spectra were recorded using an Ocean Insight Maya2000Pro spectrometer connected via an optical fiber to a Sarspec deuterium tungsten light source and a Thorlabs CVH100, cuvette holder. The measurement was performed at 25 °C.

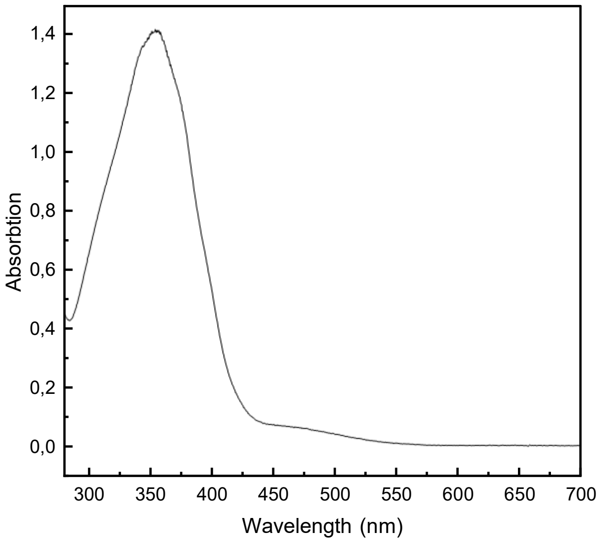

Figure S4: UV/vis absorption spectrum of (*E*)-2-(phenyldiazenyl)-4*H*-benzo[d][1,3]oxazin-4-one (**5aa**) recorded in CH_2Cl_2.

10 NMR Spectra

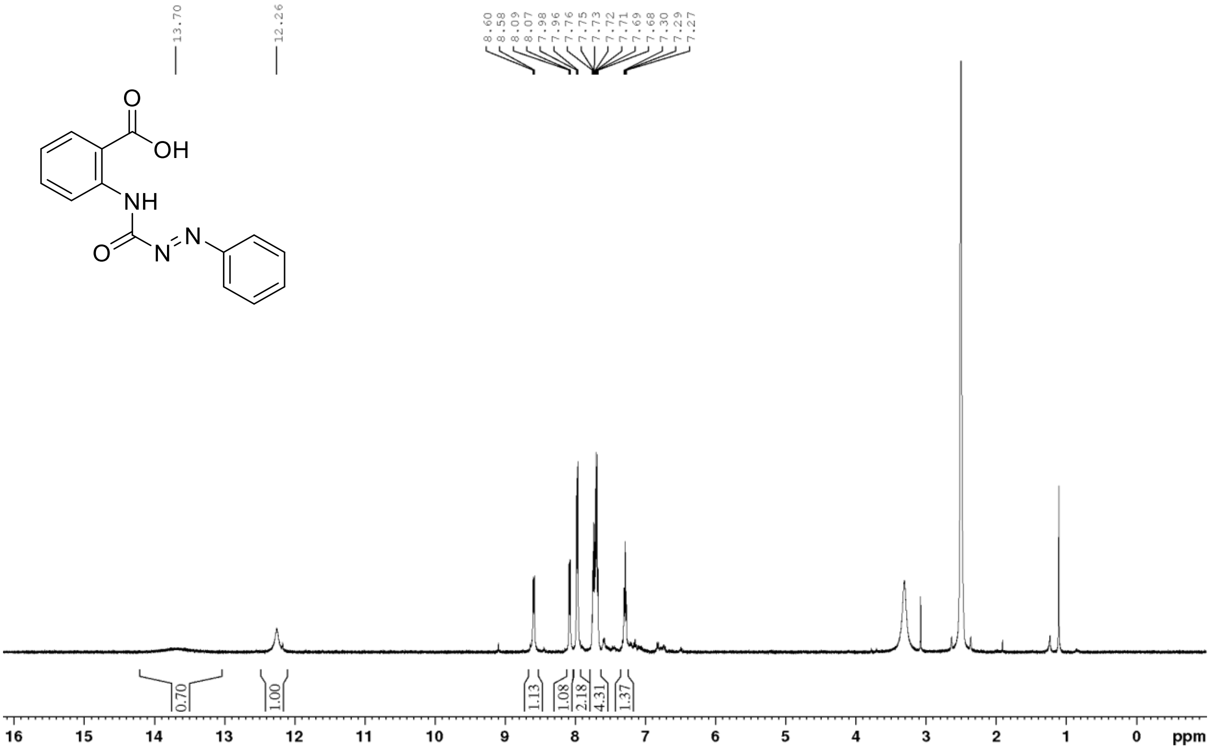

Figure S5: ^{1}H NMR (500 MHz, DMSO-d_6) of (*E*)-2-(Phenyldiazenecarboxamido)benzoic Acid (**3aa**·H).

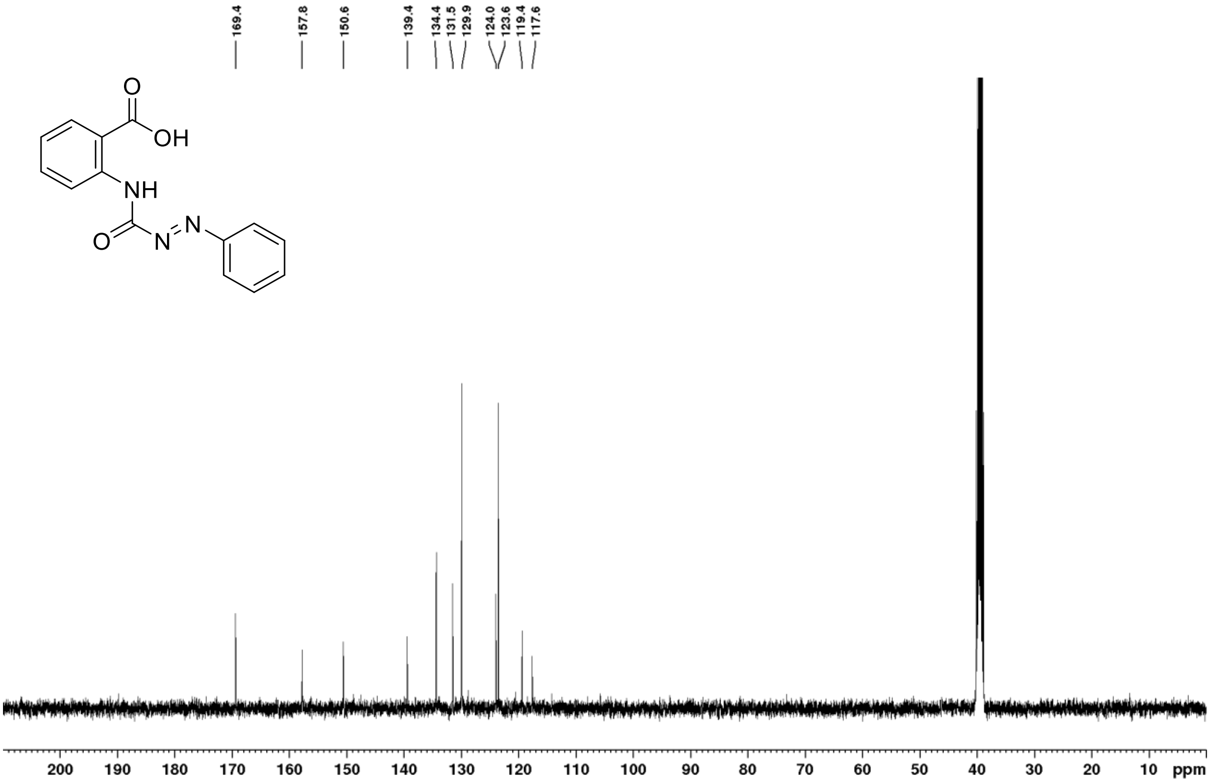

Figure S6: ^{13}C{^{1}H} NMR (101 MHz, DMSO-d_6) of (*E*)-2-(Phenyldiazenecarboxamido)benzoic Acid (**3aa**·H).

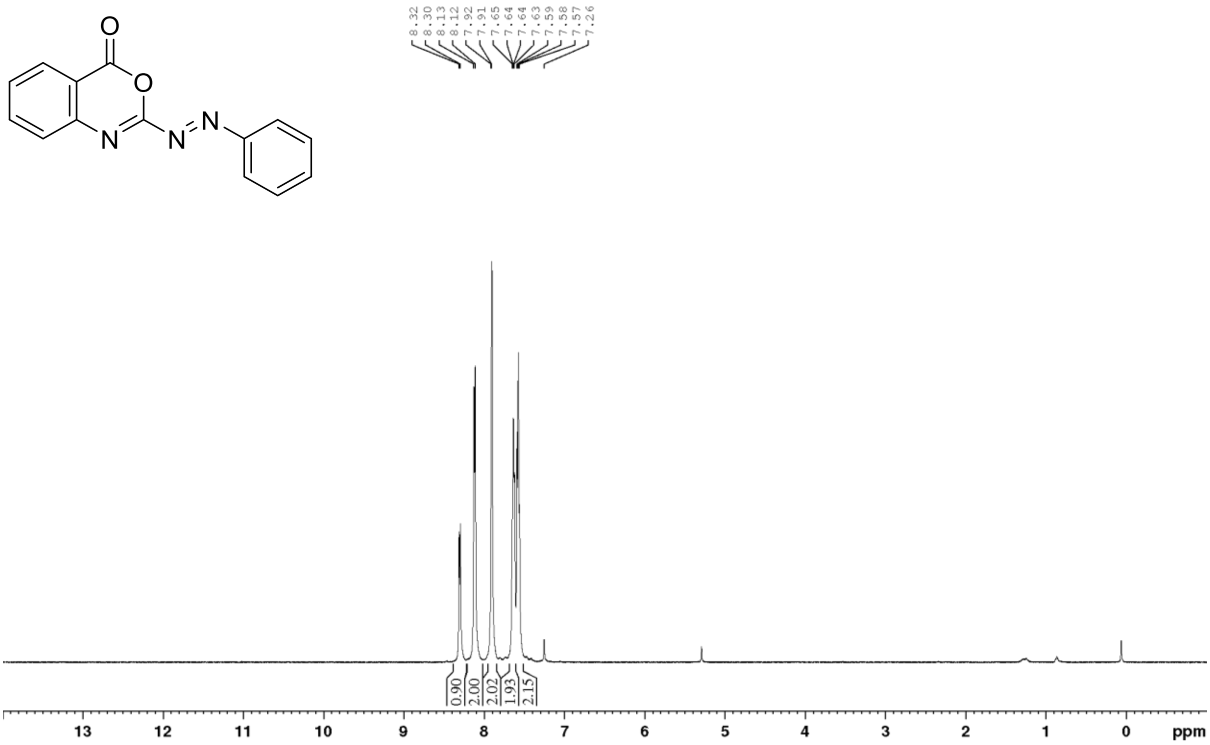

Figure S7: ^{1}H NMR (500 MHz, $CDCl_3$) of (*E*)-2-(Phenyldiazenyl)-4*H*-benzo[d][1,3]oxazin-4-one (**5aa**).

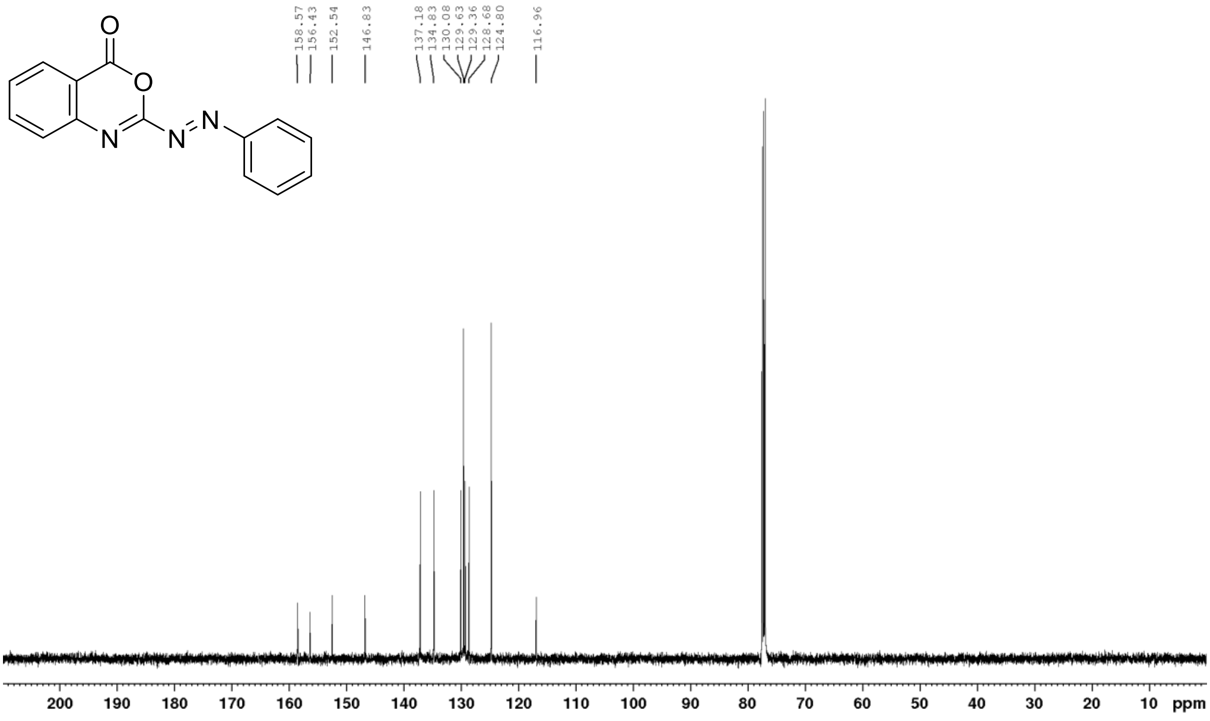

Figure S8: $^{13}C\{^{1}H\}$ NMR (126 MHz, $CDCl_3$) of (*E*)-2-(Phenyldiazenyl)-4*H*-benzo[d][1,3]oxazin-4-one (**5aa**).

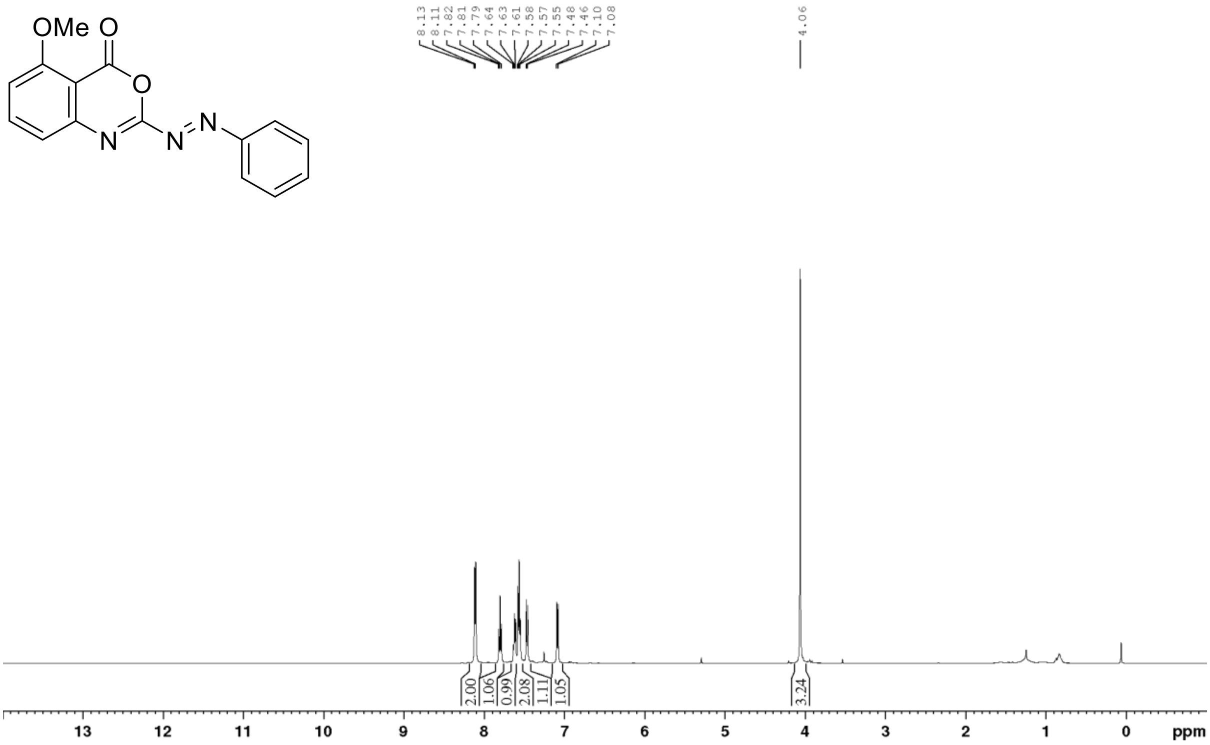

Figure S9: ^{1}H NMR (500 MHz, $CDCl_3$) of (*E*)-5-Methoxy-2-(phenyldiazenyl)-4*H*-benzo[d][1,3]oxazin-4-one (**5ba**).

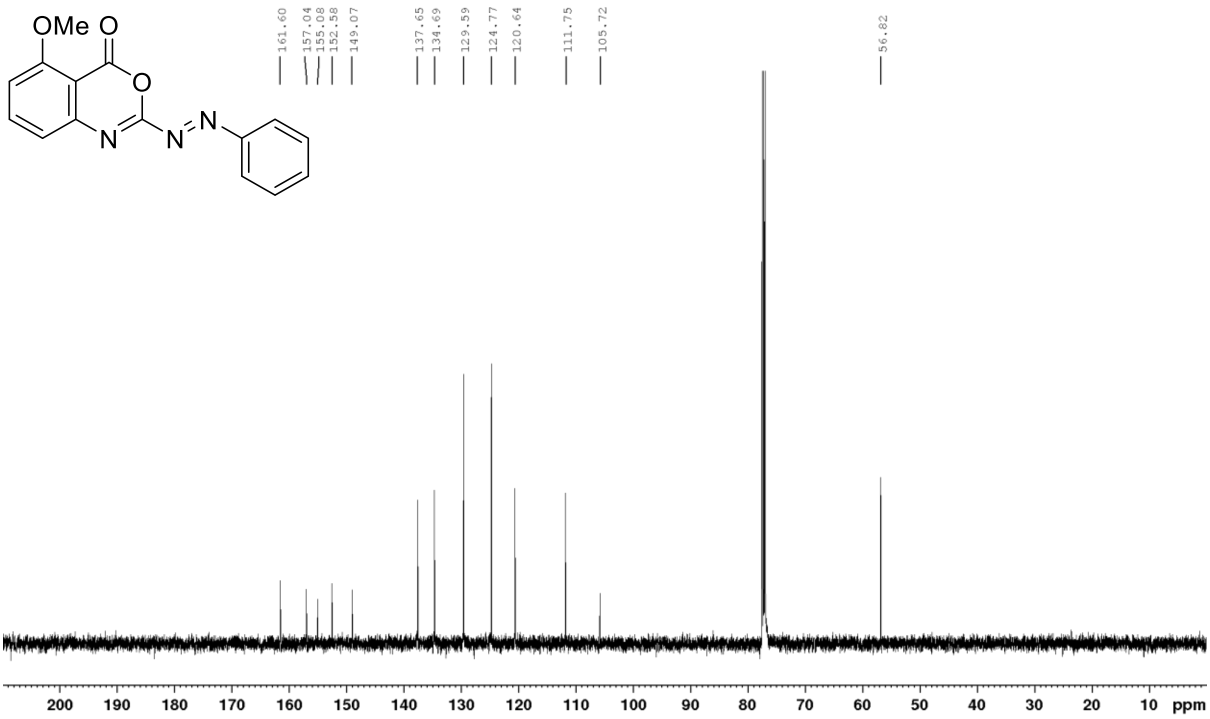

Figure S10: $^{13}C\{^{1}H\}$ NMR (126 MHz, $CDCl_3$) of (*E*)-5-Methoxy-2-(phenyldiazenyl)-4*H*-benzo[d]-[1,3]oxazin-4-one (**5ba**).

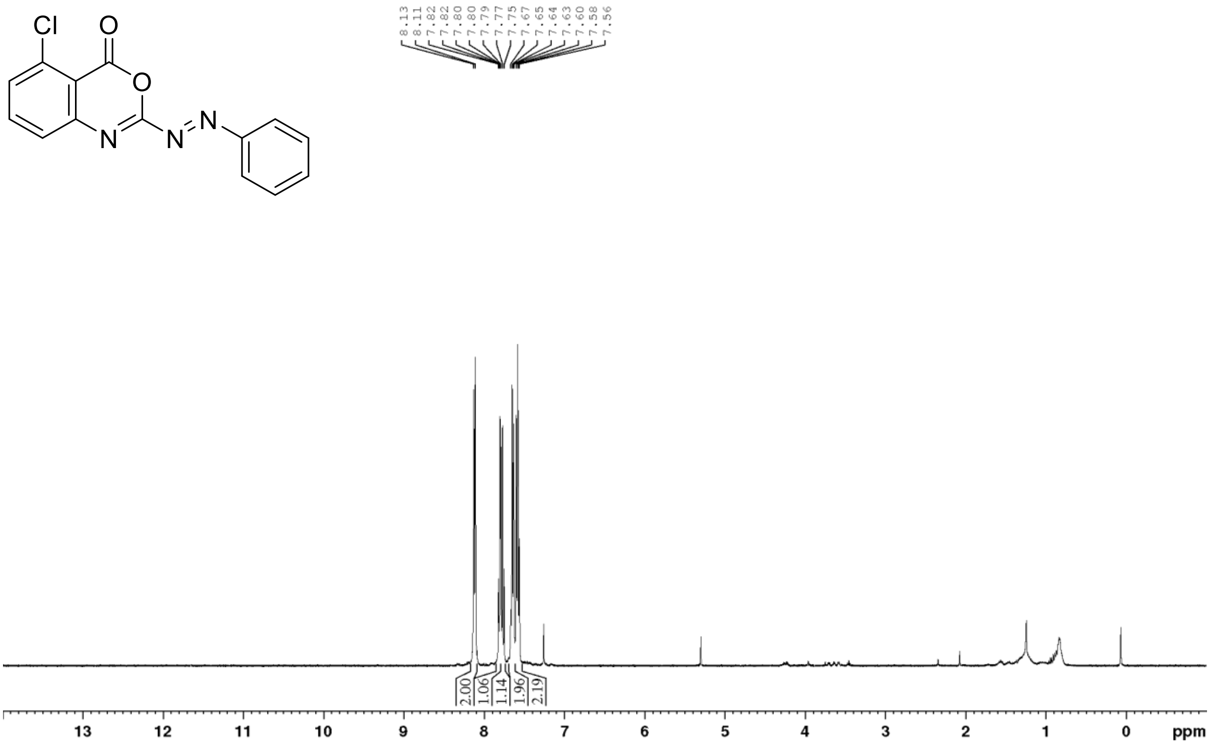

Figure S11: ^{1}H NMR (400 MHz, $CDCl_3$) of (*E*)-5-Chloro-2-(phenyldiazenyl)-4*H*-benzo[d][1,3]oxazin-4-one (**5ca**).

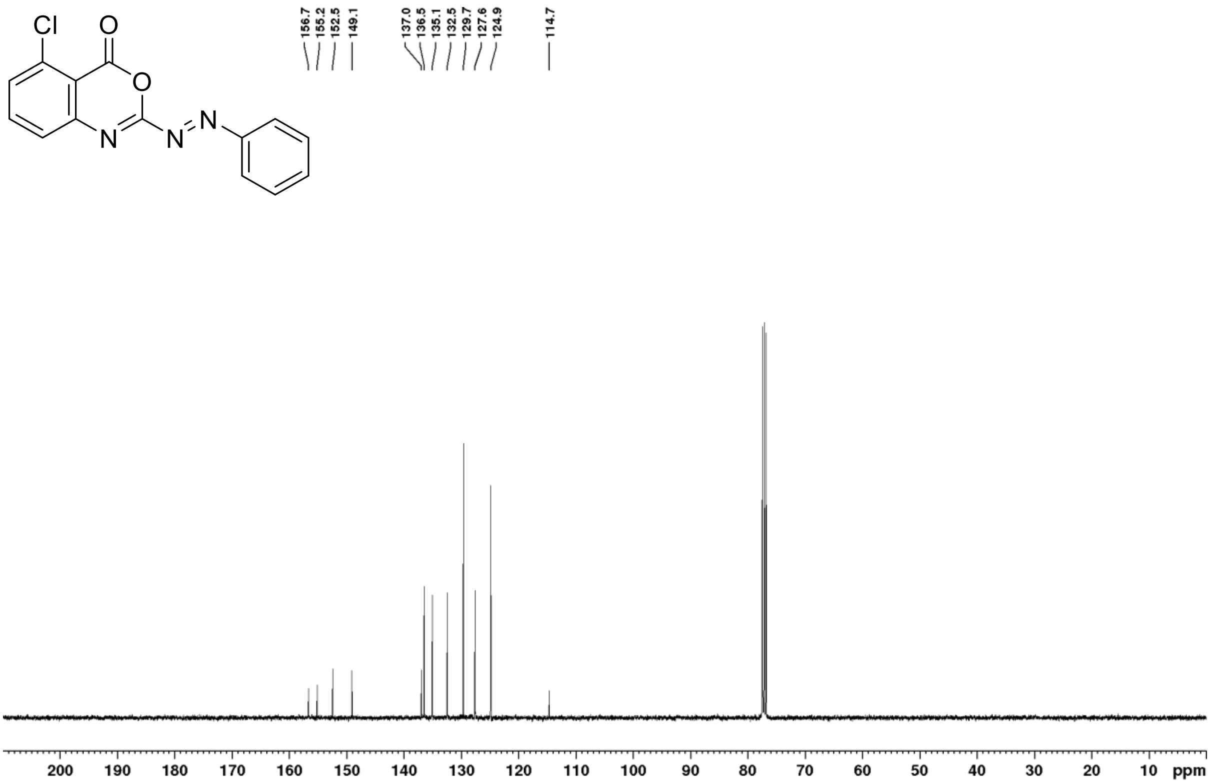

Figure S12: $^{13}C\{^{1}H\}$ NMR (101 MHz, $CDCl_3$) of (*E*)-5-Chloro-2-(phenyldiazenyl)-4*H*-benzo[d][1,3]oxazin-4-one (**5ca**).

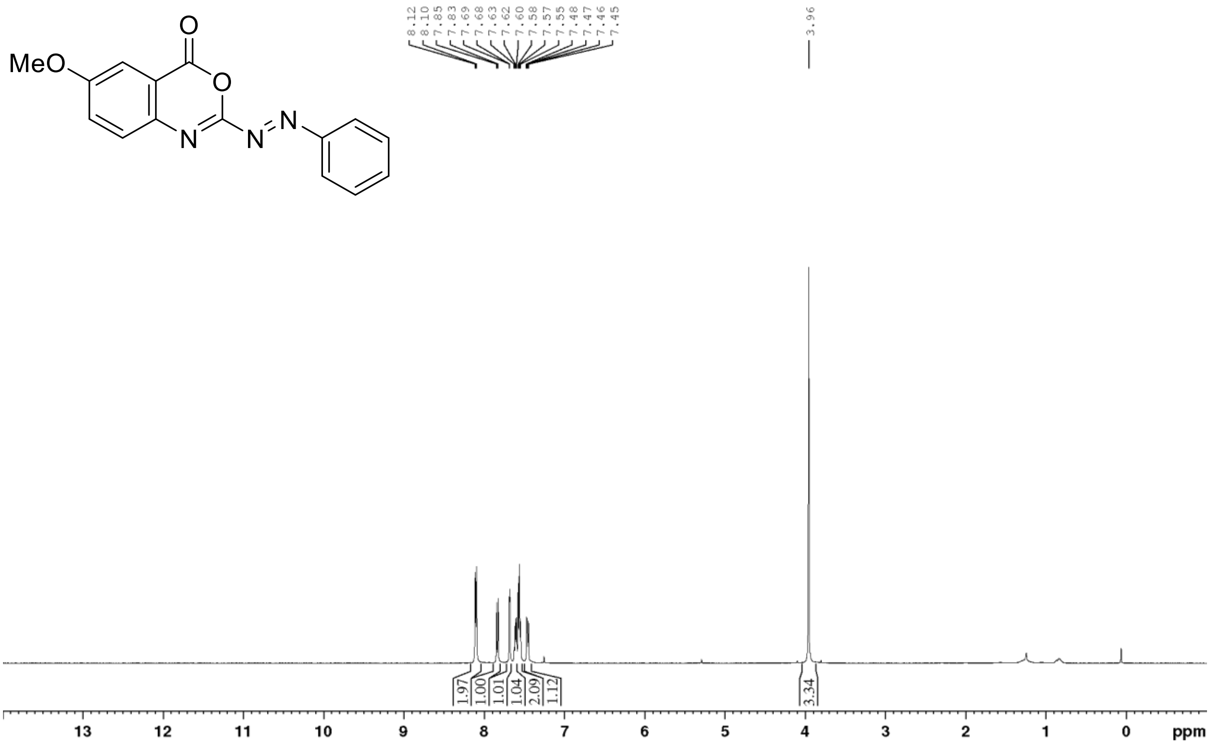

Figure S13: ^{1}H NMR (500 MHz, $CDCl_3$) of (*E*)-6-Methoxy-2-(phenyldiazenyl)-4*H*-benzo[d][1,3]oxazin-4-one (**5da**).

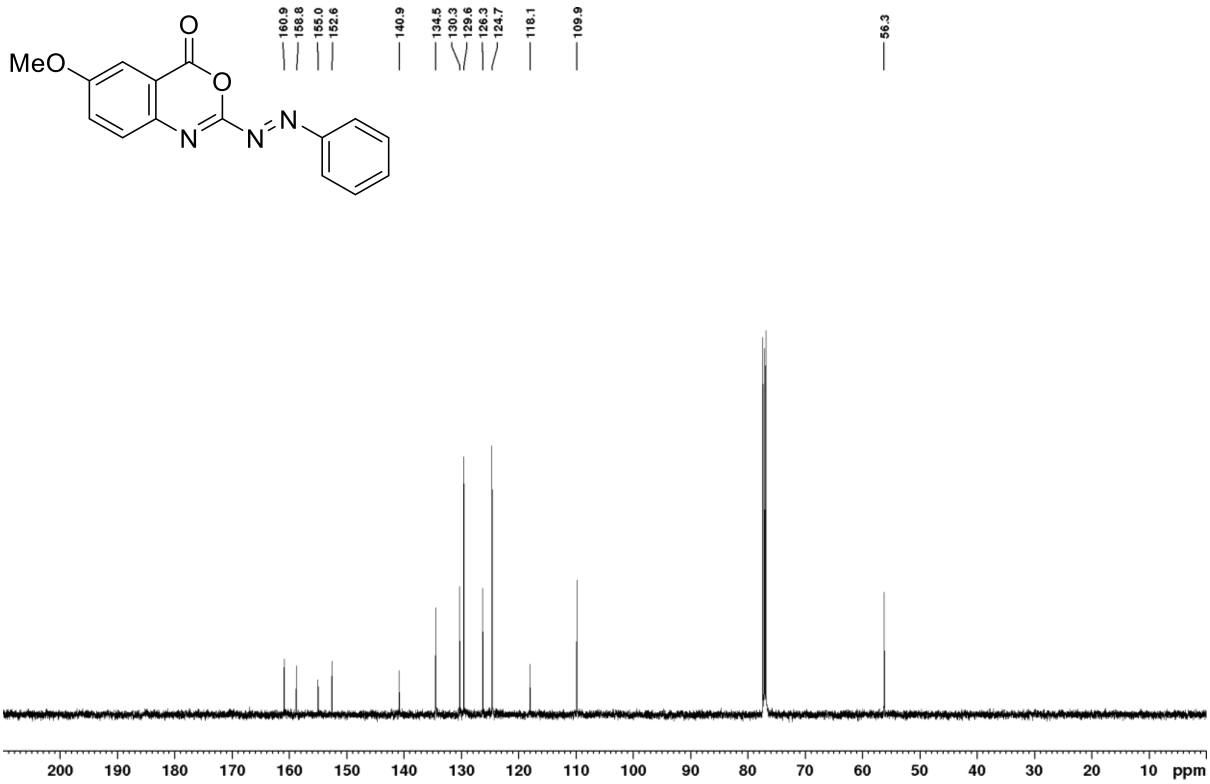

Figure S14: $^{13}C\{^{1}H\}$ NMR (126 MHz, $CDCl_3$) of (*E*)-6-Methoxy-2-(phenyldiazenyl)-4*H*-benzo[d][1,3]oxazin-4-one (**5da**).

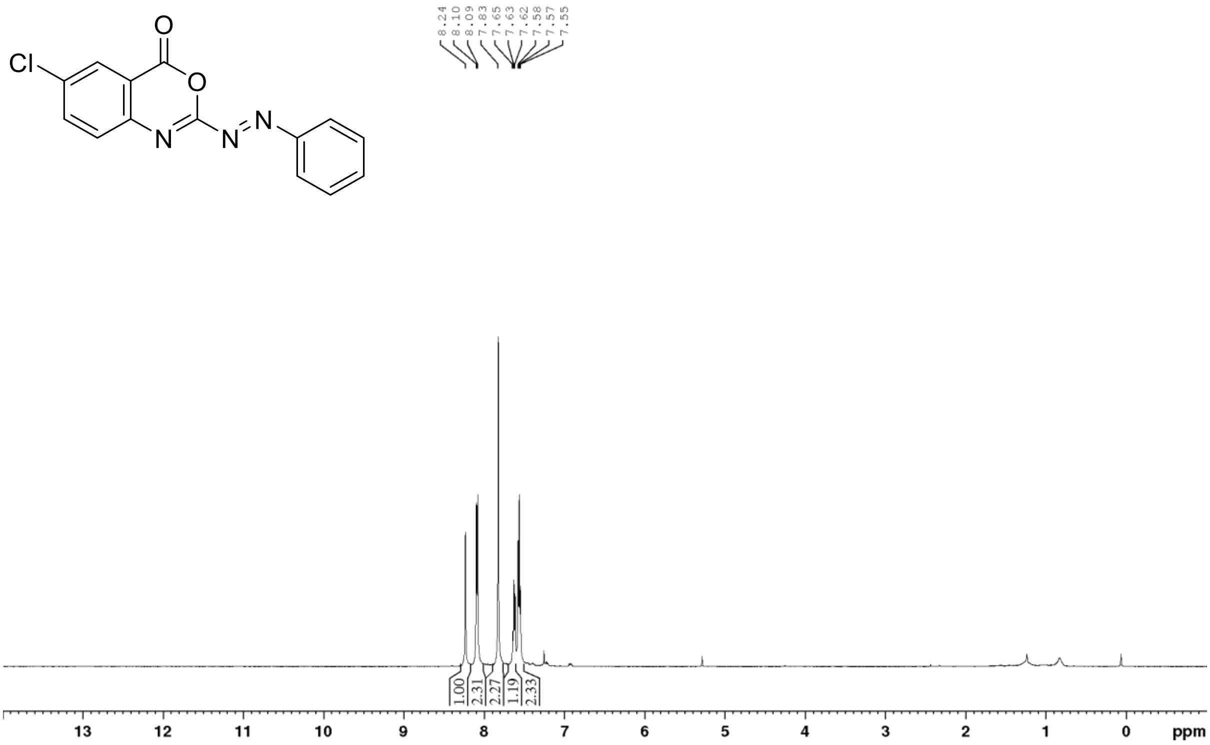

Figure S15: ^{1}H NMR (500 MHz, $CDCl_3$) of (*E*)-6-Chloro-2-(phenyldiazenyl)-4*H*-benzo[d][1,3]oxazin-4-one (**5ea**).

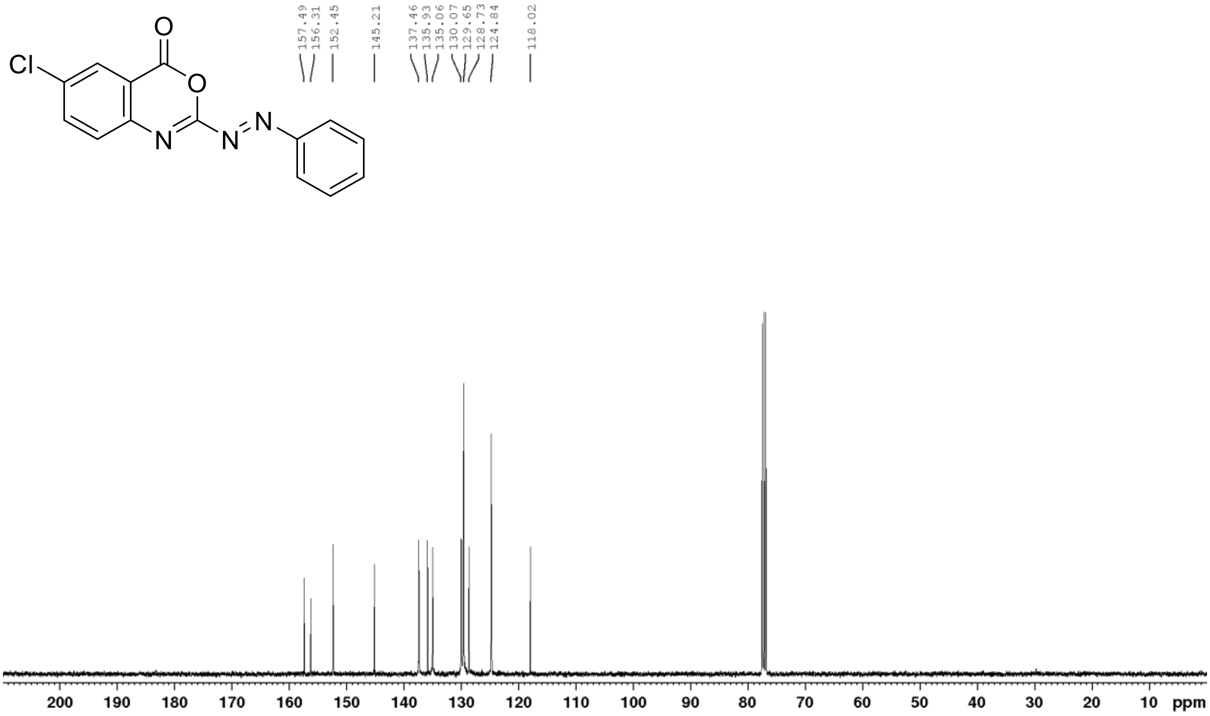

Figure S16: $^{13}C\{^{1}H\}$ NMR (101 MHz, $CDCl_3$) of (*E*)-6-Chloro-2-(phenyldiazenyl)-4*H*-benzo[d][1,3]oxazin-4-one (**5ea**).

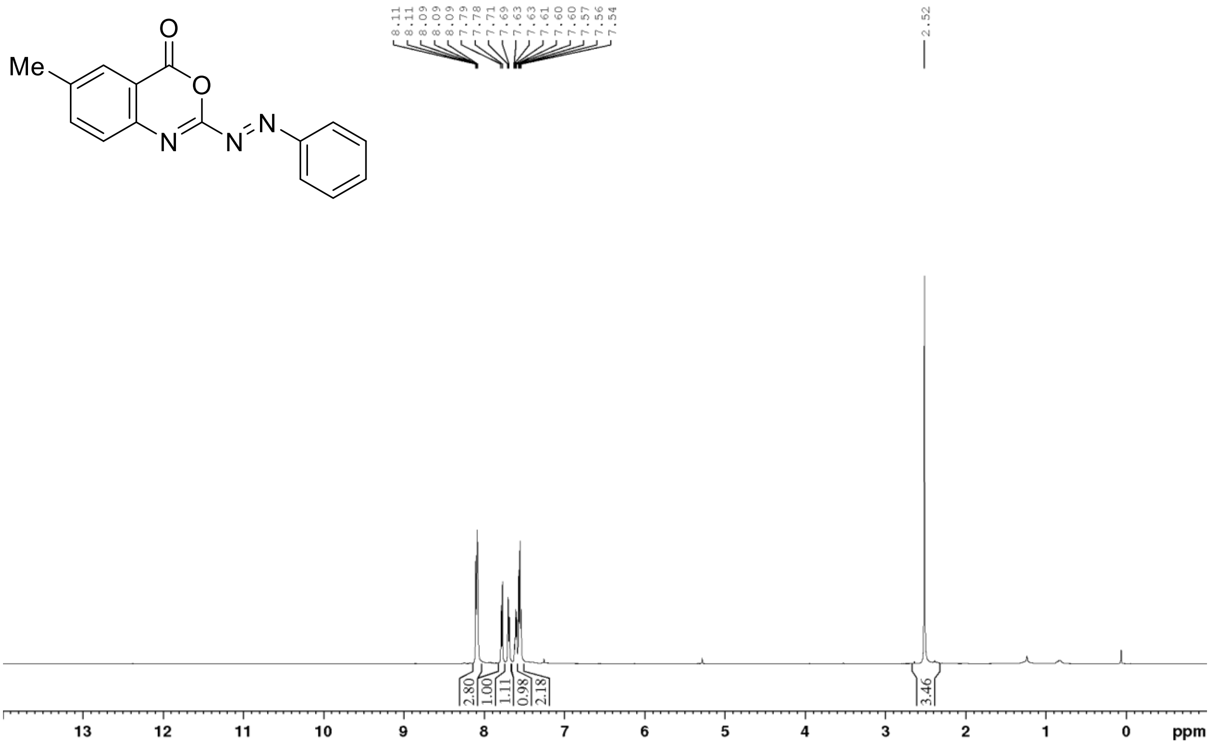

Figure S17: ^{1}H NMR (500 MHz, $CDCl_3$) of (*E*)-6-Methyl-2-(phenyldiazenyl)-4*H*-benzo[d][1,3]oxazin-4-one (**5fa**).

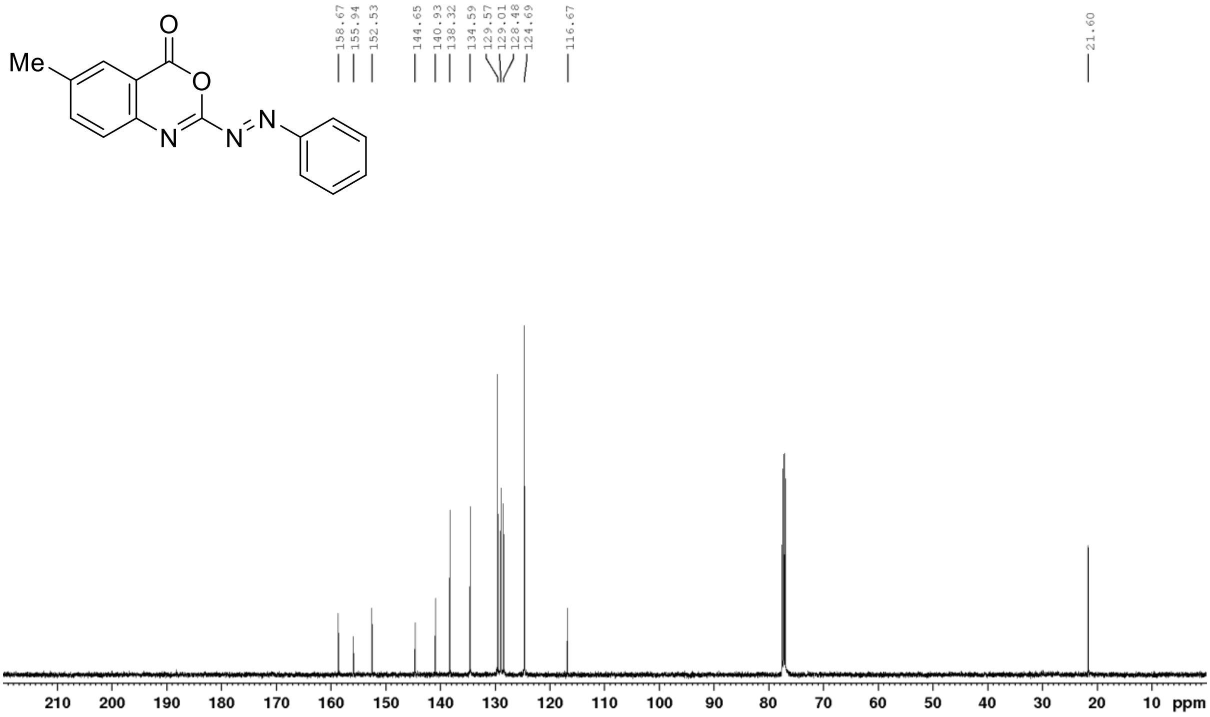

Figure S18: ^{13}C{^{1}H} NMR (126 MHz, $CDCl_3$) of (*E*)-6-Methyl-2-(phenyldiazenyl)-4*H*-benzo[d]-[1,3]oxazin-4-one (**5fa**).

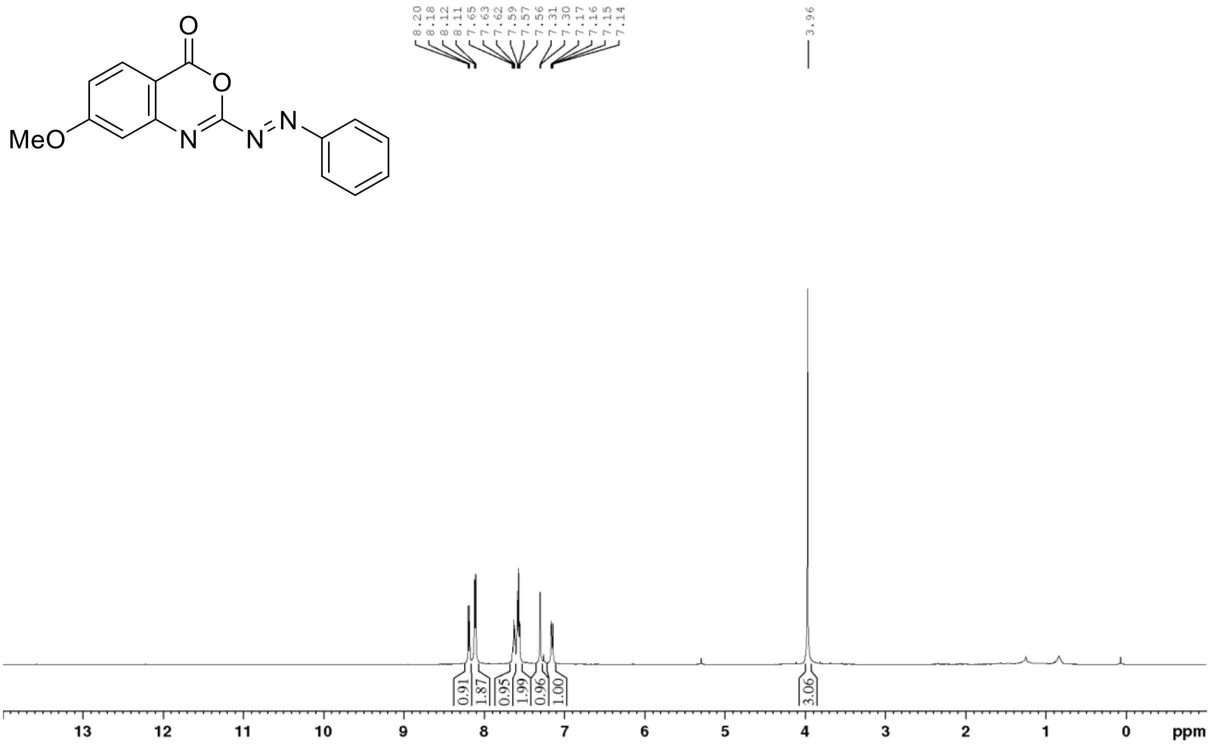

Figure S19: ^{1}H NMR (500 MHz, $CDCl_3$) of (*E*)-7-Methoxy-2-(phenyldiazenyl)-4*H*-benzo[d][1,3]oxazin-4-one (**5ga**).

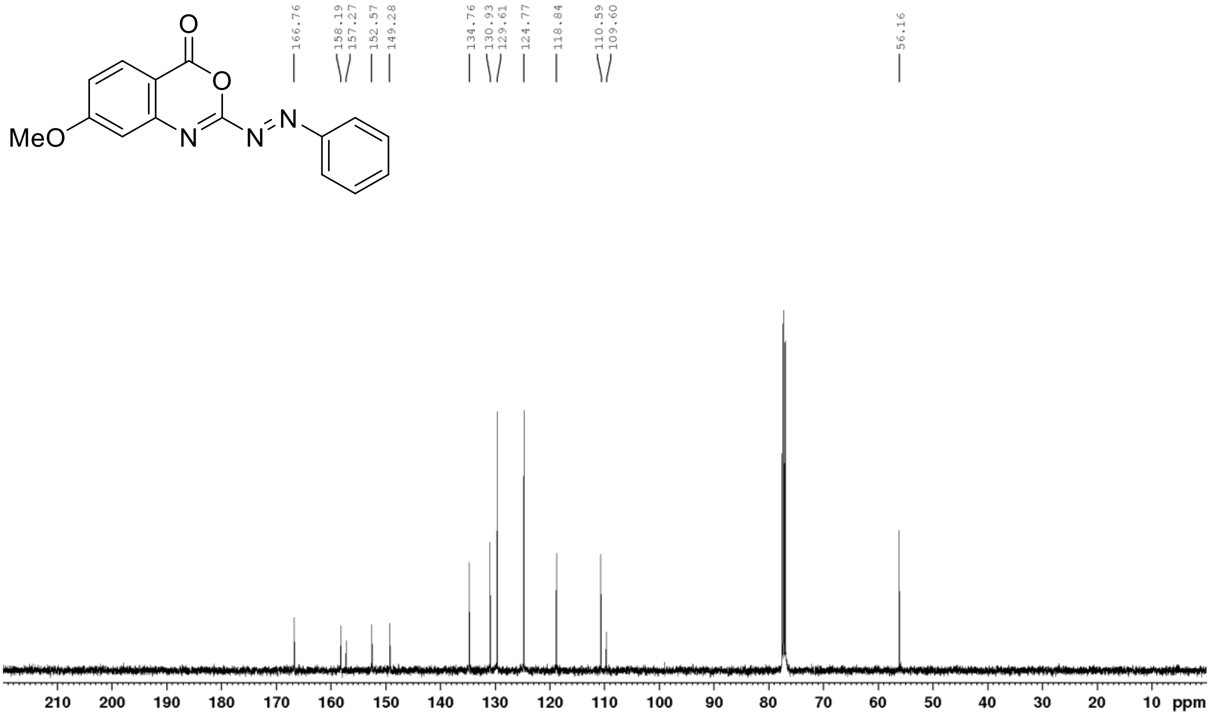

Figure S20: $^{13}C\{^{1}H\}$ NMR (126 MHz, $CDCl_3$) of (*E*)-7-Methoxy-2-(phenyldiazenyl)-4*H*-benzo[d][1,3]oxazin-4-one (**5ga**).

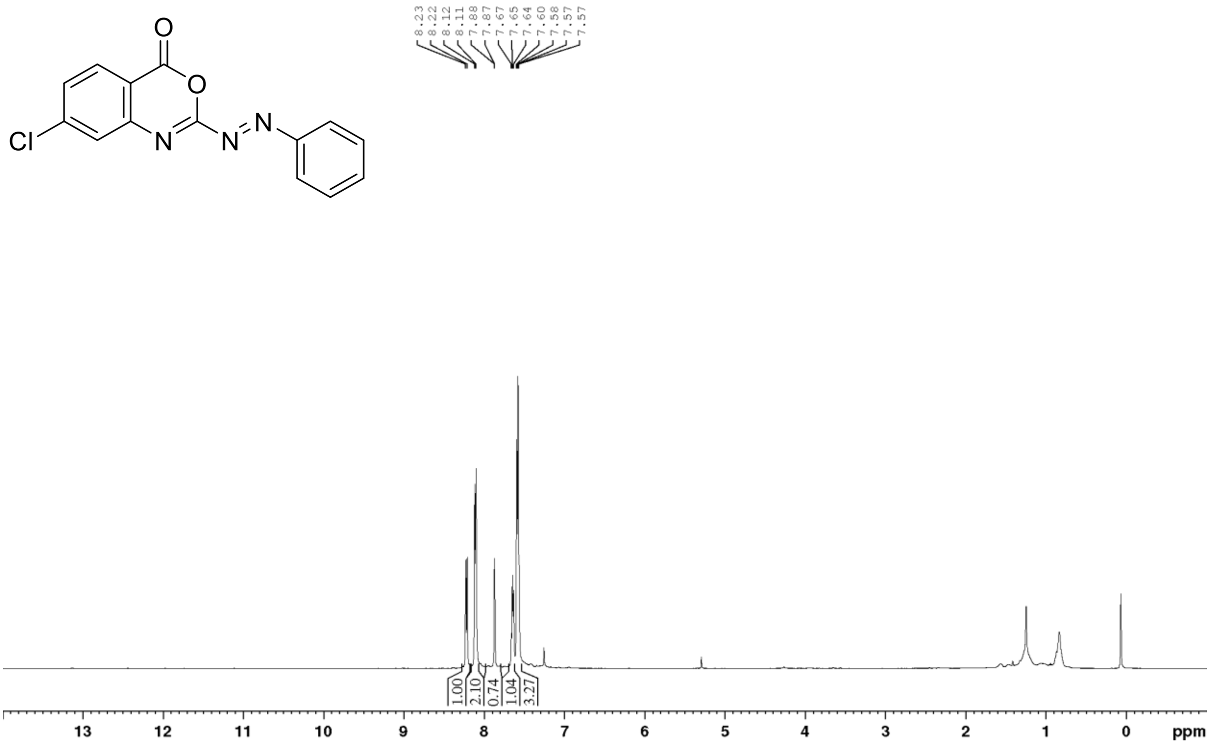

Figure S21: ^{1}H NMR (500 MHz, $CDCl_3$) of (*E*)-7-Chloro-2-(phenyldiazenyl)-4*H*-benzo[d][1,3]oxazin-4-one (**5ha**).

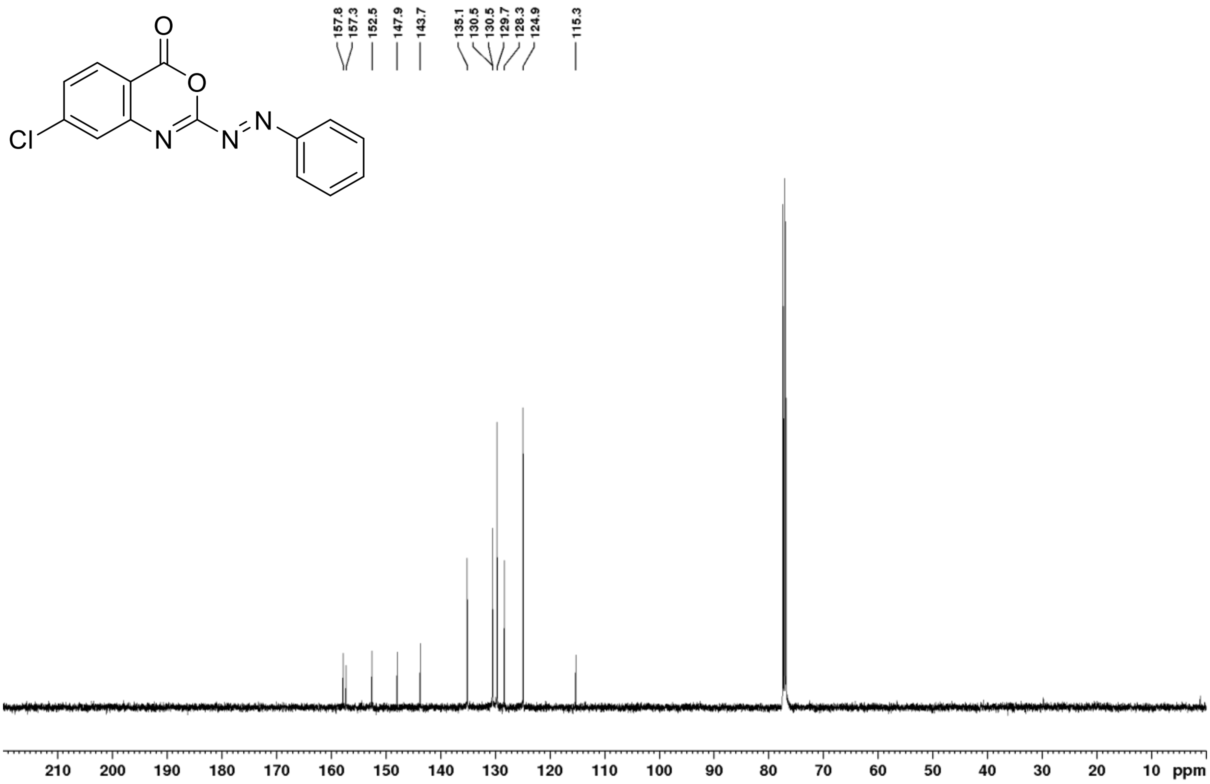

Figure S22: $^{13}C\{^{1}H\}$ NMR (126 MHz, $CDCl_3$) of (*E*)-7-Chloro-2-(phenyldiazenyl)-4*H*-benzo[d][1,3]oxazin-4-one (**5ha**).

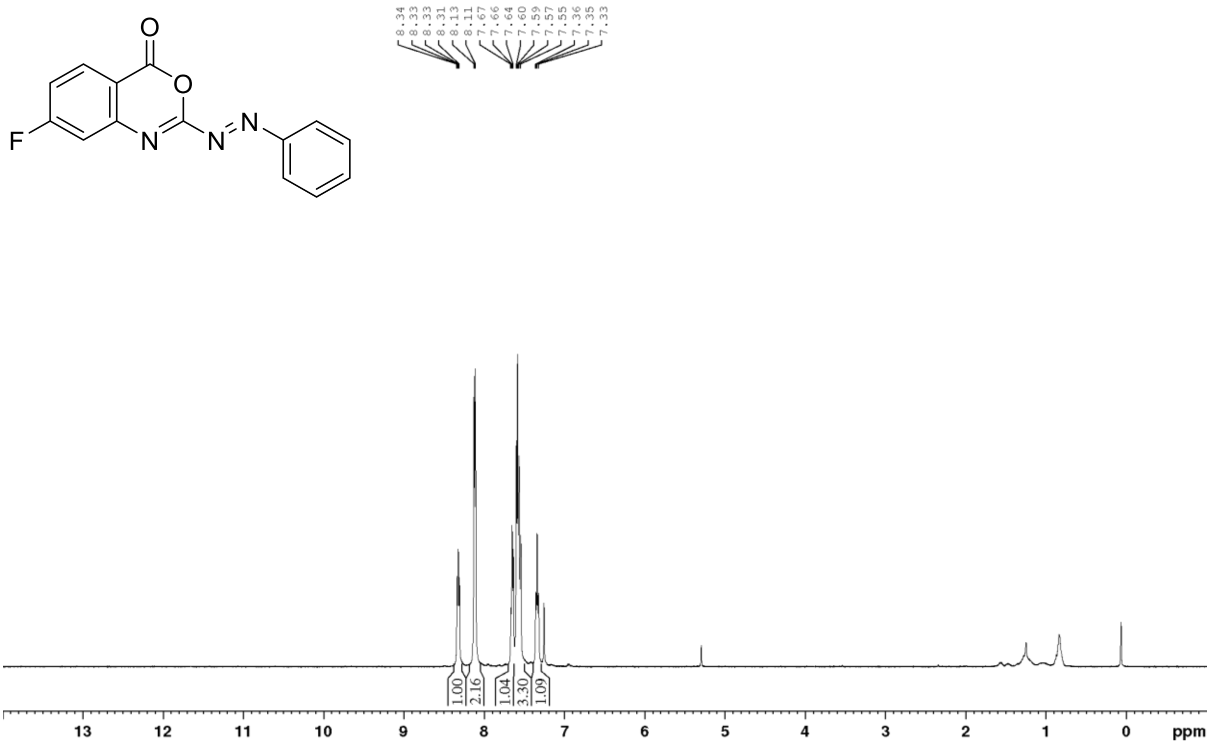

Figure S23: ^{1}H NMR (500 MHz, $CDCl_3$) of (*E*)-7-Fluoro-2-(phenyldiazenyl)-4*H*-benzo[d][1,3]oxazin-4-one (**5ia**).

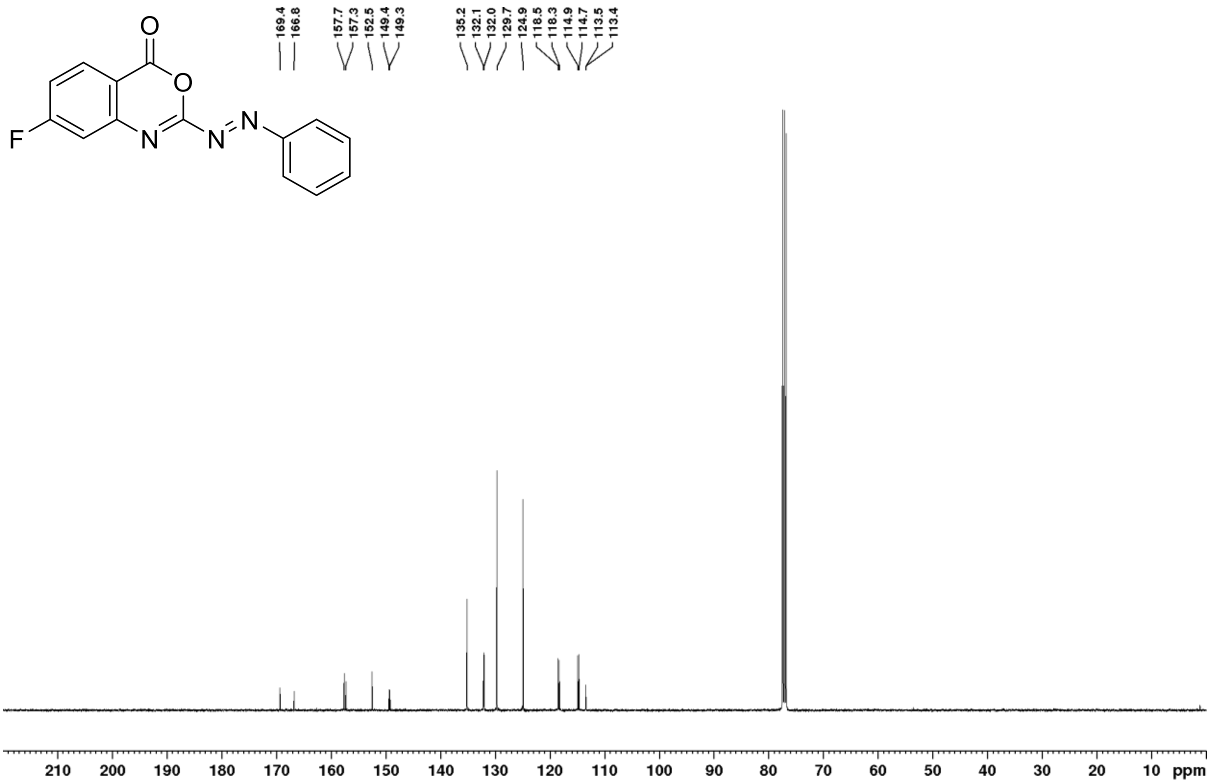

Figure S24: $^{13}C\{^{1}H\}$ NMR (101 MHz, $CDCl_3$) of (*E*)-7-Fluoro-2-(phenyldiazenyl)-4*H*-benzo[d][1,3]oxazin-4-one (**5ia**).

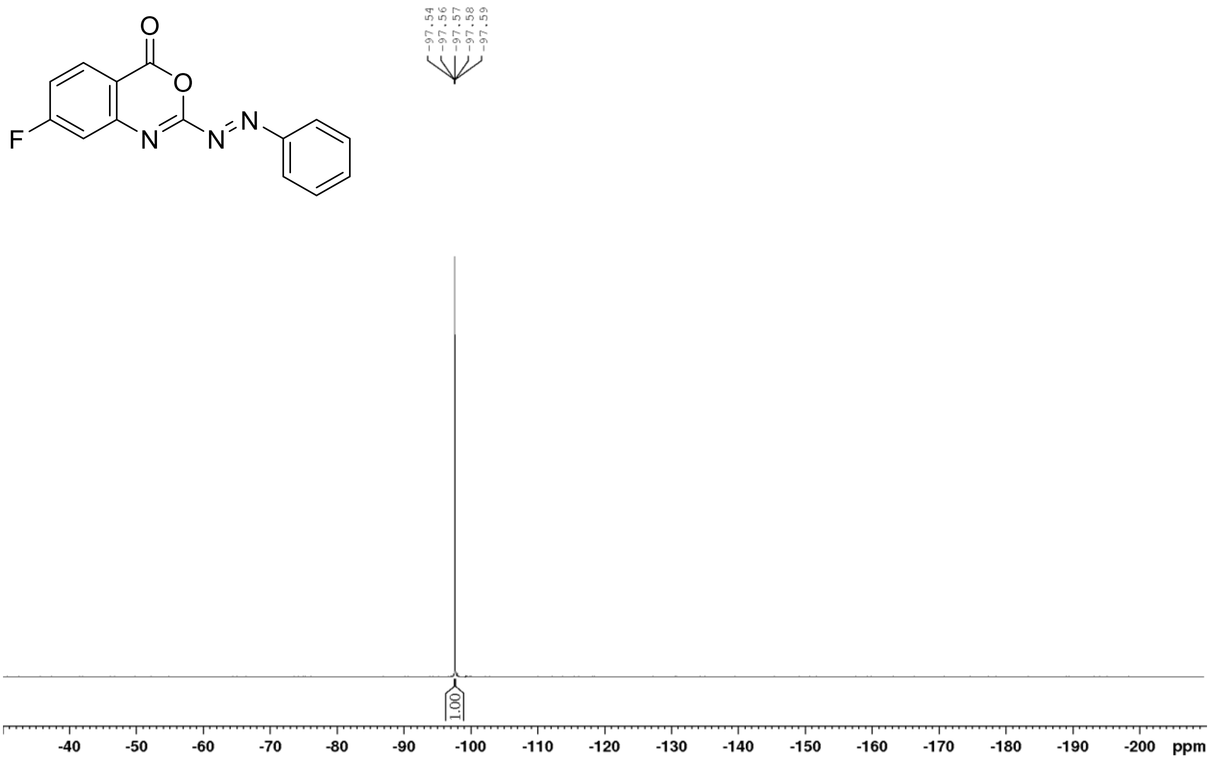

Figure S25: ^{19}F NMR (471 MHz, $CDCl_3$) of (*E*)-7-Fluoro-2-(phenyldiazenyl)-4*H*-benzo[d][1,3]oxazin-4-one (**5ia**).

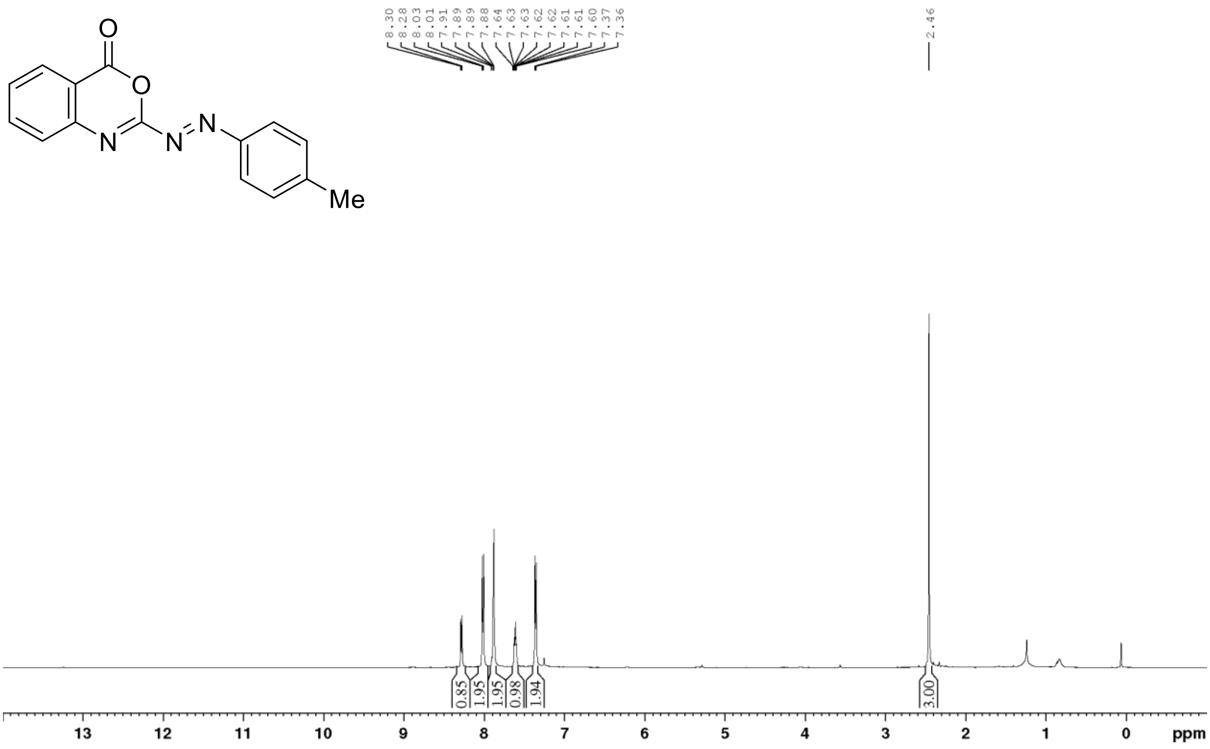

Figure S26: ^{1}H NMR (500 MHz, $CDCl_3$) of (*E*)-2-(*p*-Tolyldiazenyl)-4*H*-benzo[d][1,3]oxazin-4-one (**5ab**).

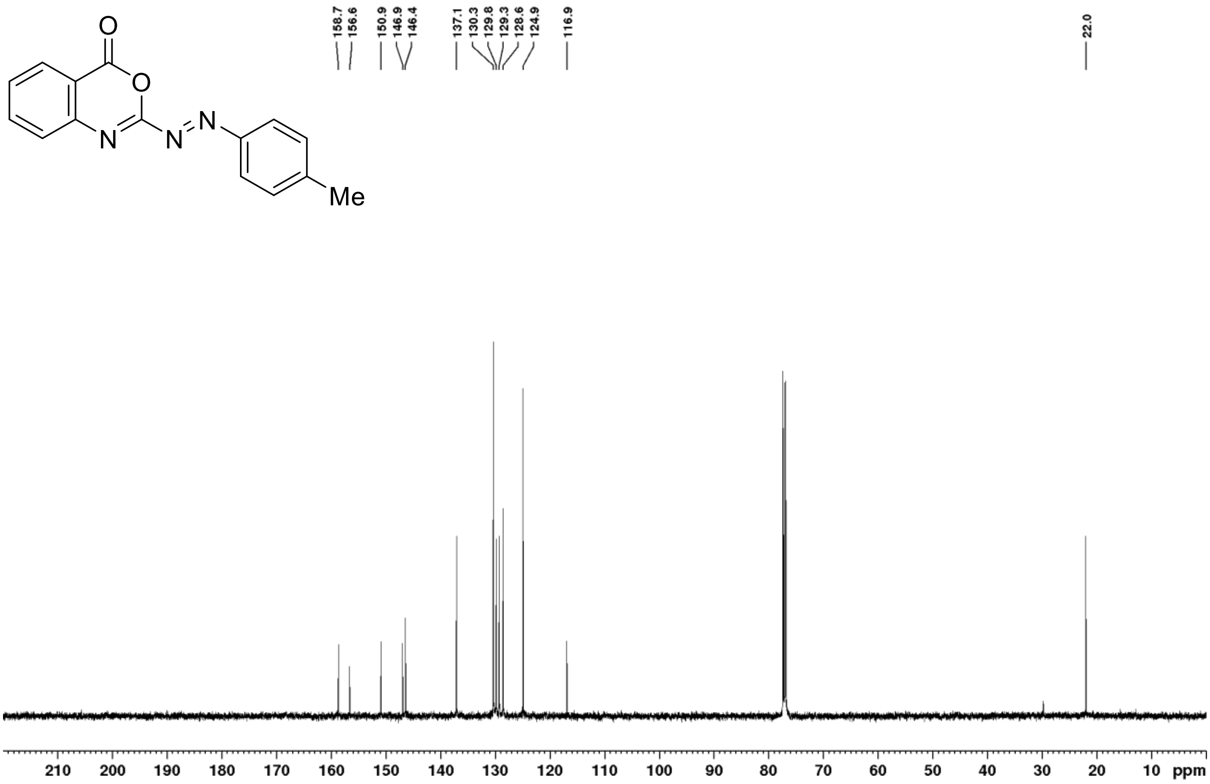

Figure S27: $^{13}C\{^{1}H\}$ NMR (126 MHz, $CDCl_3$) of (*E*)-2-(*p*-Tolyldiazenyl)-4*H*-benzo[d][1,3]oxazin-4-one (**5ab**).

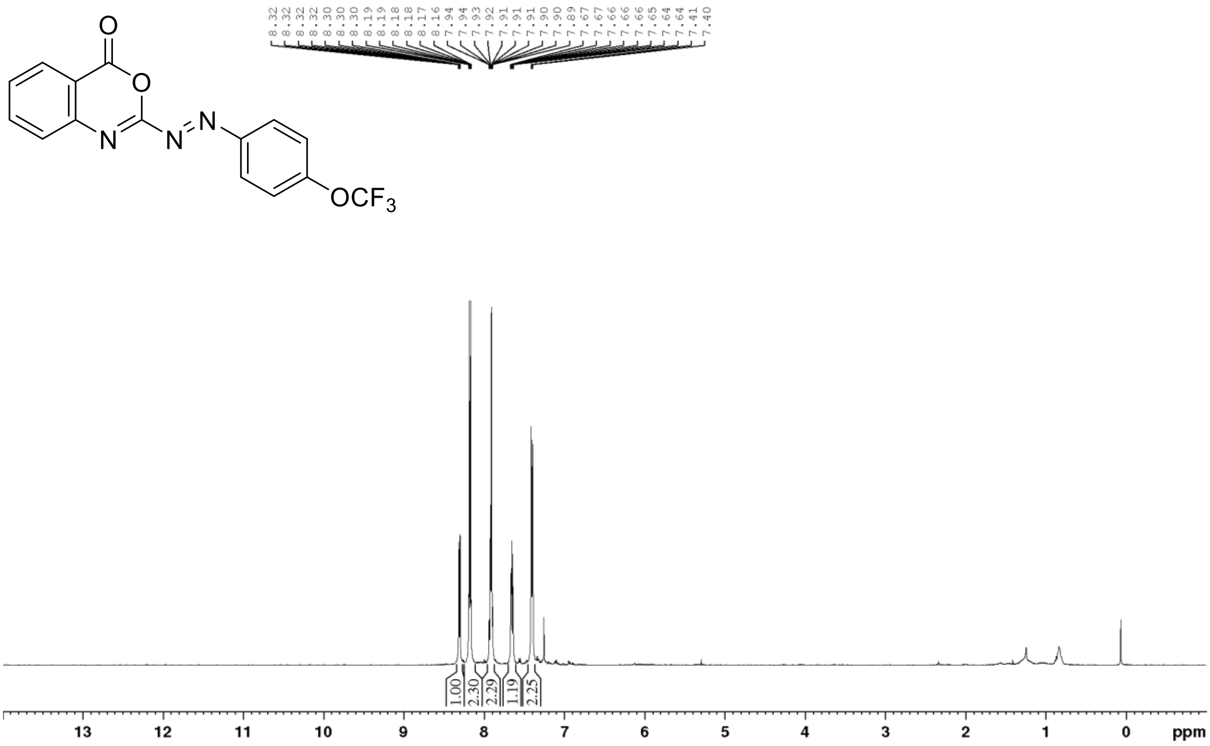

Figure S28: ^{1}H NMR (500 MHz, $CDCl_3$) of (*E*)-2-((4-(Trifluoromethoxy)phenyl)diazenyl)-4*H*-benzo[d][1,3]oxazin-4-one (**5ac**).

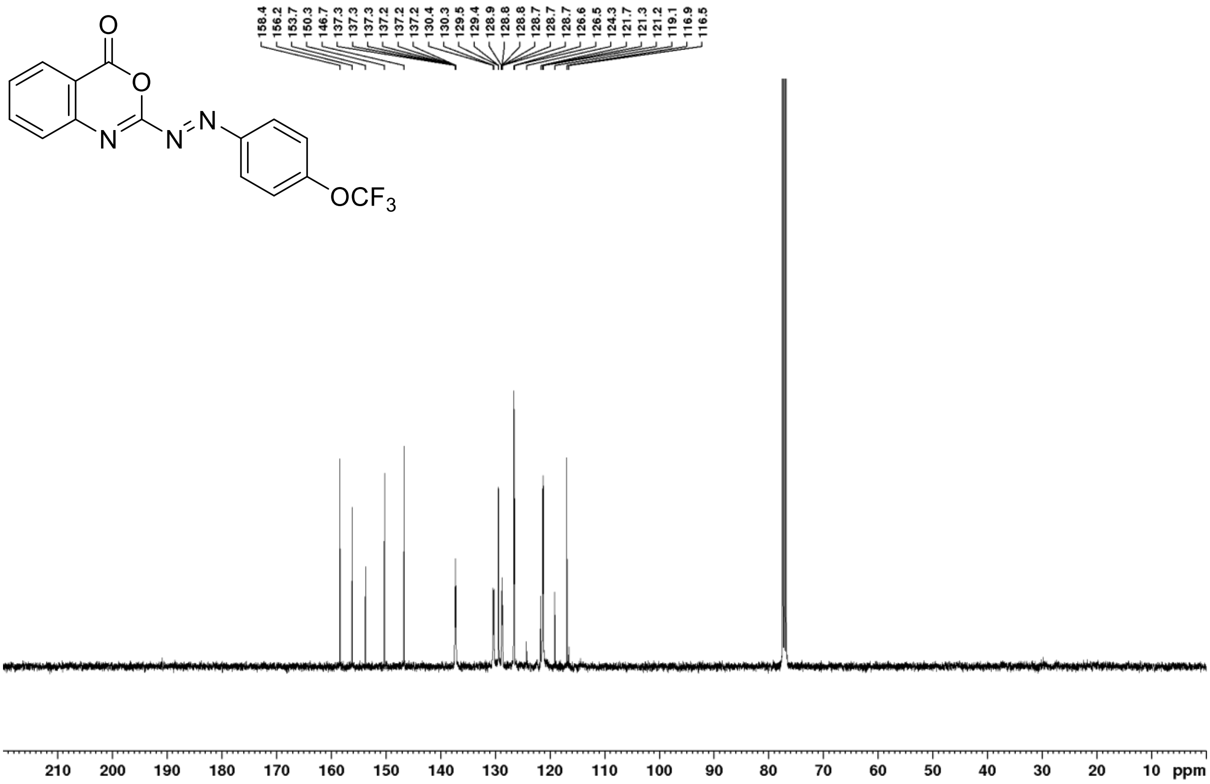

Figure S29: $^{13}C\{^{1}H\}$ NMR (101 MHz, $CDCl_3$) of (*E*)-2-((4-(Trifluoromethoxy)phenyl)diazenyl)-4*H*-benzo[d][1,3]oxazin-4-one (**5ac**).

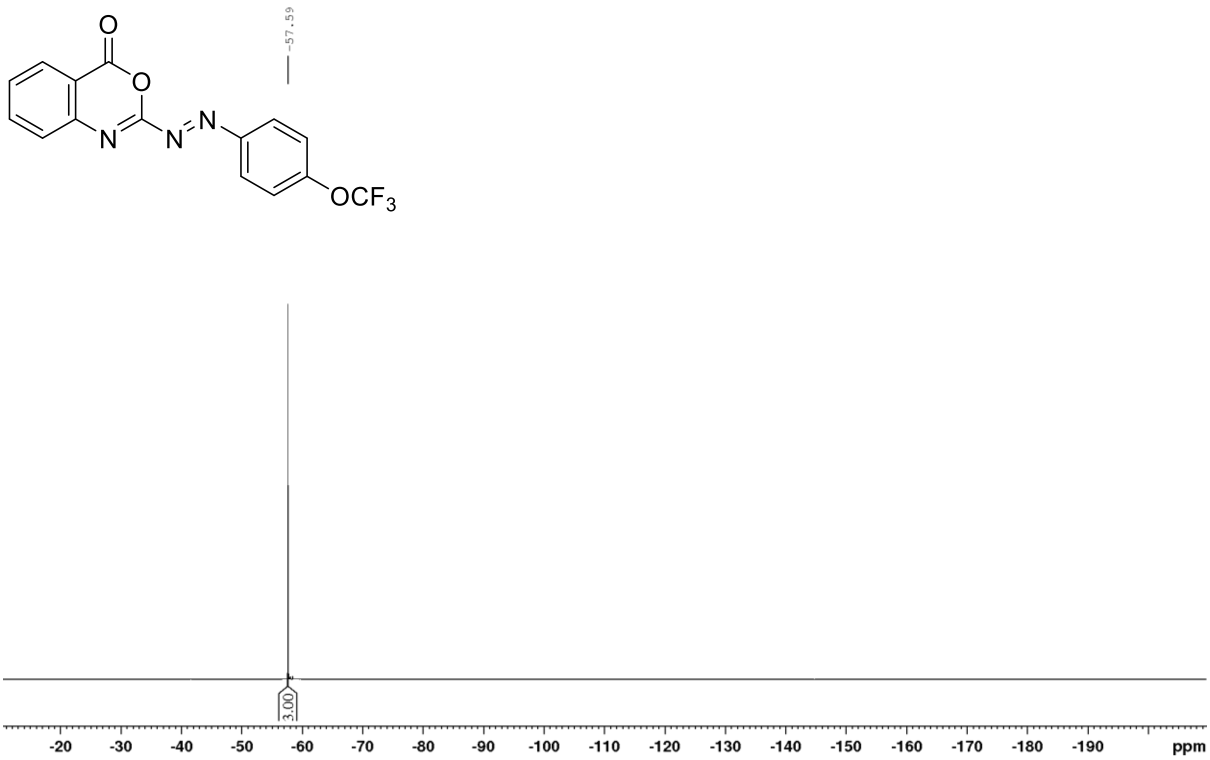

Figure S30: ^{19}F NMR (471 MHz, $CDCl_3$) of (*E*)-2-((4-(Trifluoromethoxy)phenyl)diazenyl)-4*H*-benzo[d]-[1,3]oxazin-4-one (**5ac**).

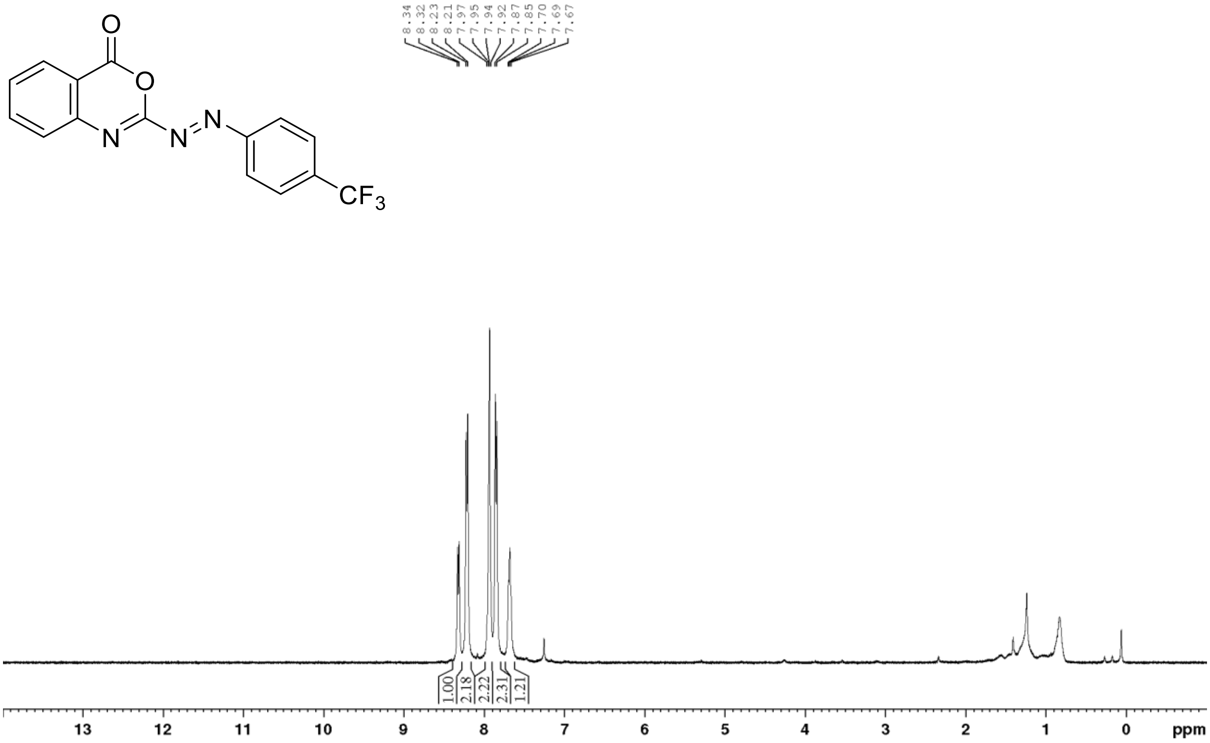

Figure S31: ^{1}H NMR (400 MHz, $CDCl_3$) of (*E*)-2-((4-(Trifluoromethyl)phenyl)diazenyl)-4*H*-benzo[d]-[1,3]oxazin-4-one (**5ad**).

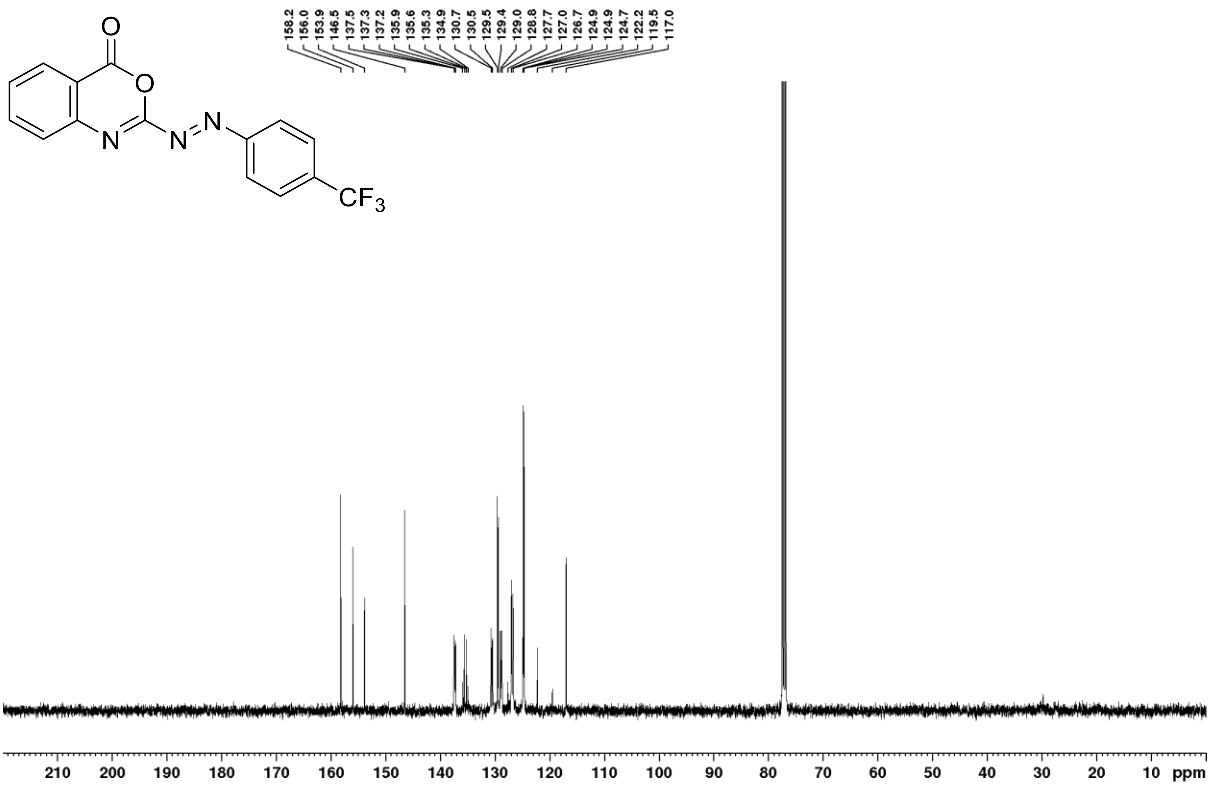

Figure S32: $^{13}C\{^{1}H\}$ NMR (101 MHz, $CDCl_3$) of (*E*)-2-((4-(Trifluoromethyl)phenyl)diazenyl)-4*H*-benzo[d][1,3]oxazin-4-one (**5ad**).

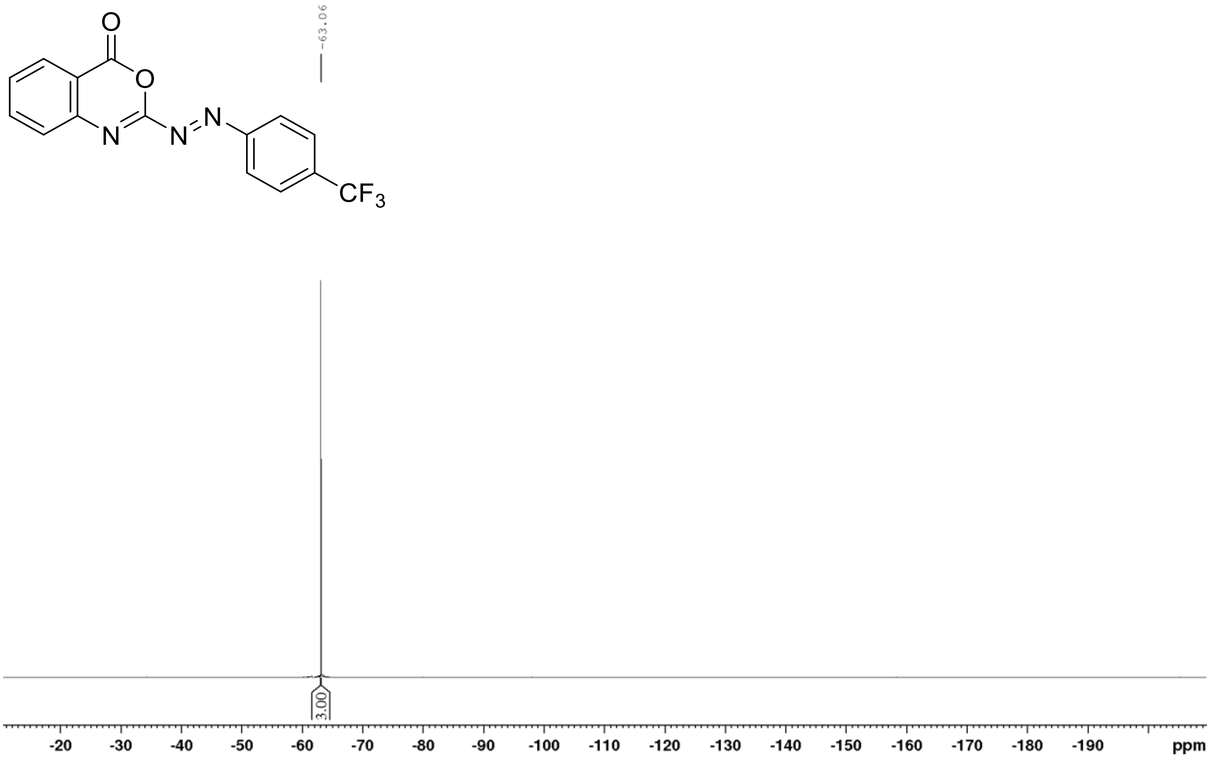

Figure S33: ^{19}F NMR (471 MHz, $CDCl_3$) of (*E*)-2-((4-(Trifluoromethyl)phenyl)diazenyl)-4*H*-benzo[d]-[1,3]oxazin-4-one (**5ad**).

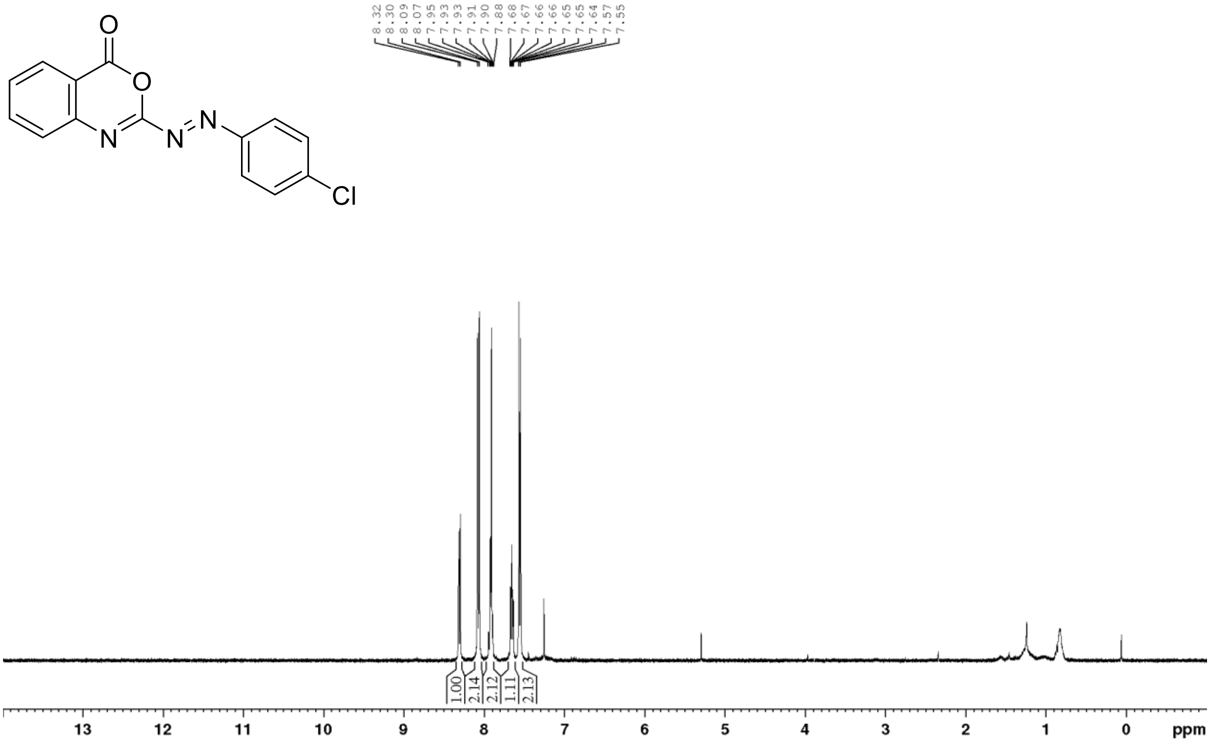

Figure S34: ^{1}H NMR (400 MHz, $CDCl_3$) of (*E*)-2-((4-Chlorophenyl)diazenyl)-4*H*-benzo[d][1,3]oxazin-4-one (**5ae**).

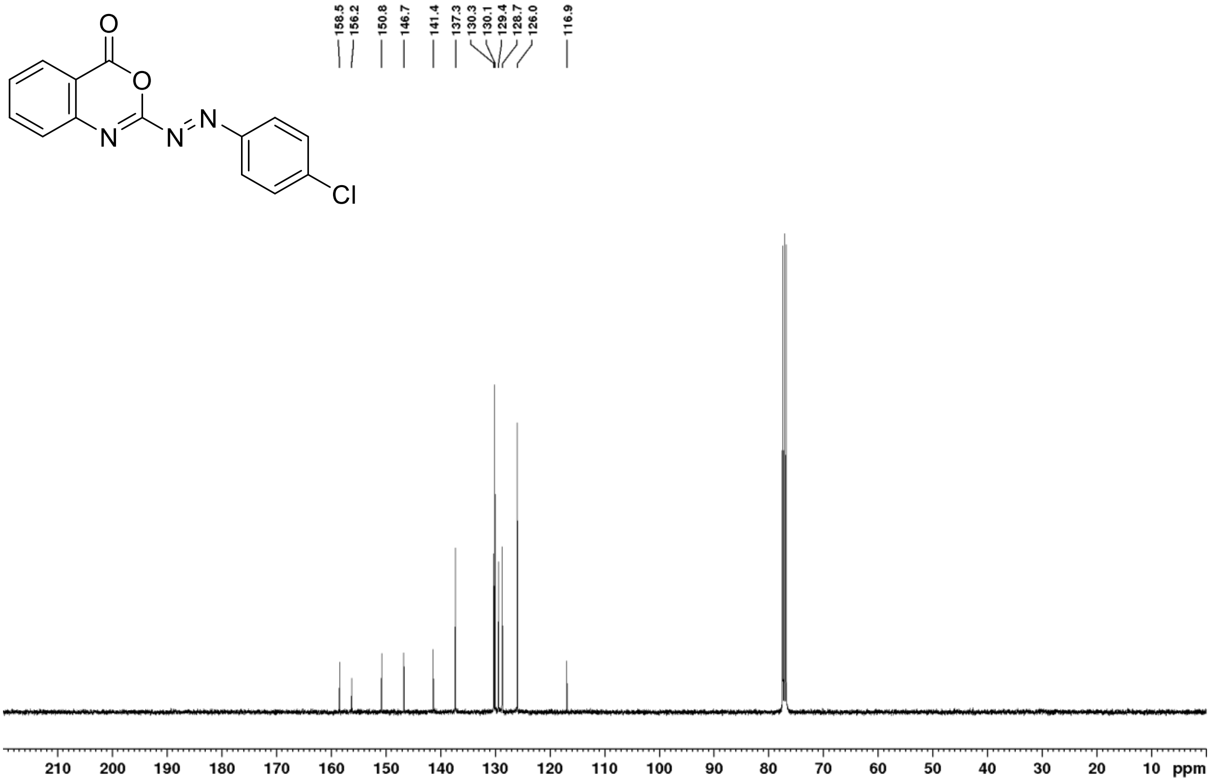

Figure S35: $^{13}C\{^1H\}$ NMR (101 MHz, $CDCl_3$) of (*E*)-2-((4-Chlorophenyl)diazenyl)-4*H*-benzo[d][1,3]oxazin-4-one (**5ae**).

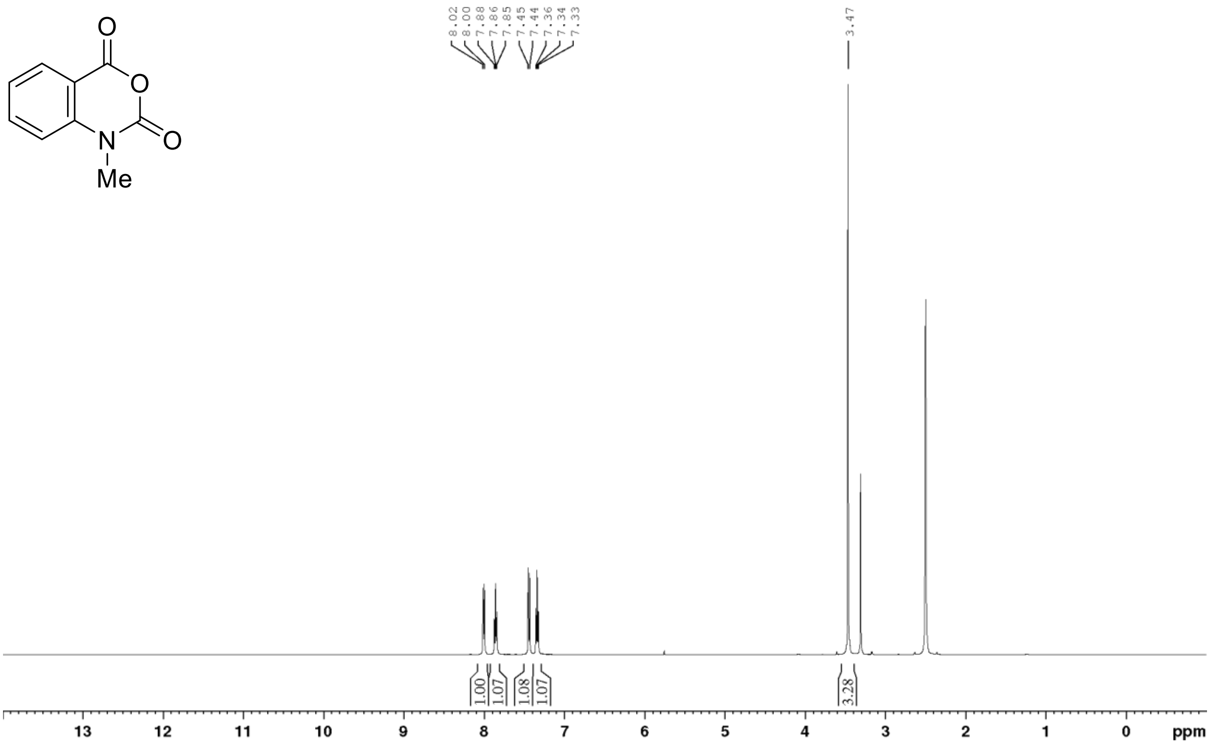

Figure S36: ^1H NMR (500 MHz, DMSO-d_6) of *N*-Methylisatoic Anhydride (**9**).

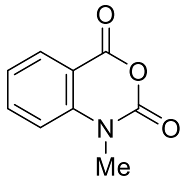

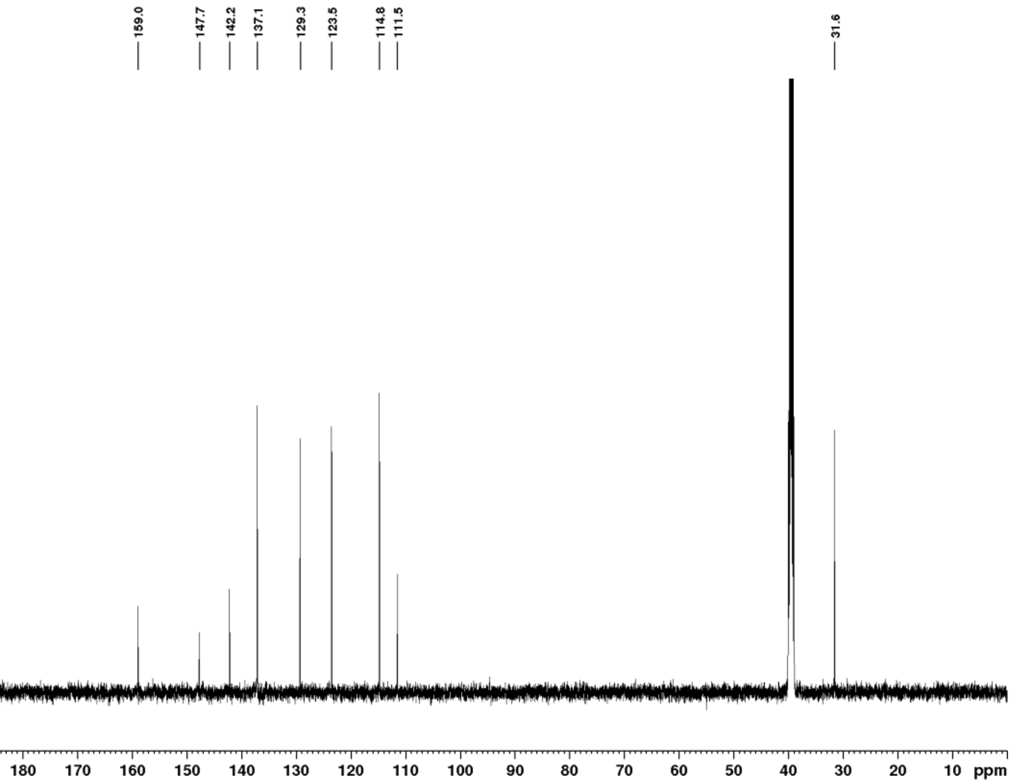

Figure S37: $^{13}C\{^1H\}$ NMR (126 MHz, DMSO-d_6) of *N*-Methylisatoic Anhydride (**9**).

11 References

S1 Chauvier, C.; Finck, L.; Hecht S.; Oestreich M. General Synthesis and Optical Properties of *N*-Aryl-*N′*-Silyldiazenes. *Organometallics* **2019**, *38*, 4679–4686.

S2 Guan, Z. H.; Rean, Z. H. Palladium-Catalyzed Regioselective Carbonylation of C–H Bonds of N-Alkyl Anilines for Synthesis of Isatoic Anhydrides. *J. Am. Chem. Soc.* **2012**, *134*, 17490–17493.

S3 Liu, X.; Li, H.-Q.; Ye, S.; Liu, Y.-M.; He, H.-Y.; Cao, Y. Gold-Catalyzed Direct Hydrogenative Coupling of Nitroarenes to Synthesize Aromatic Azo Compounds. *Angew. Chem., Int. Ed.* **2014**, *53*, 7624–7628.

S4 Agilent CrysAlisPro, Data Collection and Processing Software for Agilent X-ray Diffractometers, **2012**, Agilent Technologies, Yarnton, UK.

S5 Sheldrick, G. M. Phase annealing in *SHELX*-90: Direct methods for larger structures. *Acta Crystallogr., Sect. A* **1990**, *46*, 467–473.

S6 Sheldrick, G. M. A short history of SHELX. *Acta Crystallogr., Sect. A* **2008**, *64*, 112–122.

S7 Cambridge Crystallographic Data Centre, Mercury 2024.3.0, Cambridge, UK, **2024**, can be found under https://www.ccdc.cam.ac.uk/solutions/software/mercury/.
